# Melatonin as a Topical/Systemic Formulation for the Management of Periodontitis: A Systematic Review

**DOI:** 10.3390/ma14092417

**Published:** 2021-05-06

**Authors:** Thodur Madapusi Balaji, Saranya Varadarajan, Raghunathan Jagannathan, Jaideep Mahendra, Hammam Ibrahim Fageeh, Hytham N. Fageeh, Shazia Mushtaq, Hosam Ali Baeshen, Shilpa Bhandi, Archana A. Gupta, A. Thirumal Raj, Rodolfo Reda, Shankaragouda Patil, Luca Testarelli

**Affiliations:** 1 Department of Dentistry, Bharathiraja Hospital and Research Institute, Chennai 600017, India; tmbala81@gmail.com; 2 Department of Oral Pathology and Microbiology, Sri Venkateswara Dental College and Hospital, Chennai 600130, India; vsaranya87@gmail.com (S.V.); thirumalraj666@gmail.com (A.T.R.); 3 Department of Periodontology, Tagore Dental College and Hospital, Chennai 600127, India; doctorraghunathan@gmail.com; 4 Department of Periodontology, Meenakshi Ammal Dental College, Meenakshi Academy of Higher Education and Research, Chennai 600095, India; jaideep_m_23@yahoo.co.in; 5 Department of Preventive Dental Science, College of Dentistry, Jazan University, Jazan 45412, Saudi Arabia; hafageeh@jazanu.edu.sa (H.I.F.); hfageeh@jazanu.edu.sa (H.N.F.); 6 Dental Health Department, College of Applied Medical Sciences, King Saud University, Riyadh 11362, Saudi Arabia; smushtaqdr@gmail.com; 7 Department of Orthodontics, College of Dentistry, King Abdulaziz University, Jeddah 21589, Saudi Arabia; Habaeshen@kau.edu.sa; 8 Department of Restorative Dental Sciences, Division of Operative Dentistry, College of Dentistry, Jazan University, Jazan 45412, Saudi Arabia; shilpa.bhandi@gmail.com; 9 Department of Oral Pathology and Microbiology, Dr. D. Y. Patil Dental College and Hospital Dr. D. Y. Patil Vidyapeeth, Pune 411018, India; archanaanshumangupta@gmail.com; 10 Department of Oral and Maxillofacial Sciences, Sapienza University of Rome, 00161 Rome, Italy; rodolforeda17@gmail.com (R.R.); luca.testarelli@uniroma1.it (L.T.); 11 Department of Maxillofacial Surgery and Diagnostic Sciences, Division of Oral Pathology, College of Dentistry, Jazan University, Jazan 45412, Saudi Arabia

**Keywords:** melatonin, periodontitis, pocket depth, systemic, topical

## Abstract

Objectives: To qualitatively and quantitatively review the use of melatonin as a topical/systemic formulation for the management of periodontitis. Materials and methods: PubMed; Scopus; and Web of Science databases were searched using the MesH terms “melatonin” and “periodontitis”. Title and abstracts were screened to eliminate irrelevant and duplicate articles. The full text data of the screened articles were assessed using the selection criteria. Results: Of 176 identified articles (PubMed-66; Scopus-56; Web of Science-52; Cross-reference-2), only 12 studies qualified to be included in the systematic review. Four studies assessed the independent effect of 1% topical melatonin formulation while 8 articles assessed the adjunctive use of systemic melatonin formulation (1–10 mg) following scaling and root planing (SRP). All studies showed an improvement in periodontal parameters such as pocket depth, clinical attachment loss, periodontal disease index, community periodontal index, gingival bleeding scores, and prognostic marker levels in saliva and serum. A meta-analysis of data from 2 studies revealed that 1–2 mg (systemic) melatonin supplementation reduced pocket depth; although the difference was not statistically significant and hence cannot be interpreted or used for conclusive evidence. Risk of Bias Assessment tool (RoBANS) and Cochrane Collaboration RoB tool elicited a high risk of bias in the included studies. GRADE (recommendation assessment, development, and evaluation) inferred a weak recommendation for the use of melatonin in periodontitis management. Conclusions: Melatonin supplementation (topical and systemic) in periodontitis patients improved key periodontal parameters including pocket depth and clinical attachment loss. Clinical relevance: Melatonin could be a potential host modulatory agent for periodontitis management; although the data from the present review should be interpreted carefully due to the associated high risk of bias.

## 1. Introduction

Complete dental plaque and calculus removal form the basis of periodontal therapy as pathogens from plaque are initiators of periodontal disease [1]. However, in addition to scaling and root planing (SRP) and periodontal surgery, other adjunctive measures have been employed, such as the use of antimicrobials [2], antioxidants [3], anti-inflammatory agents [4], and antiseptics [5]. These measures have shown promising periodontal benefits [5]. In this connection, melatonin has been tested for the management of periodontitis [6,7,8].

Melatonin is an indoleamine molecule that is a by-product of tryptophan metabolism [9]. The pineal gland principally synthesizes and releases melatonin into the blood using enzyme machinery during the dark phase of the day [10]. Several other tissues can produce melatonin and are termed extra-pineal sites of melatonin biosynthesis. These include gingival tissues [11] and salivary glands in the oral cavity [12]. Melatonin performs numerous functions such as circadian cycle regulation [13], antioxidant defense [14], immunomodulation [15], cancer prevention [16], and regulation of bone turnover [17]. In the oral cavity, melatonin is regarded as a potent antioxidant and immunomodulatory agent [18]. Melatonin has been assayed in saliva [19], gingival crevicular fluid (GCF) [20], and gingival tissues [18], and its overall levels were found to be lowered in patients with periodontitis than healthy controls. A recently published systematic review performed by some of us, found a depletion of salivary melatonin levels in patients with periodontitis versus healthy individuals [21]. This finding is significant as it highlights the need for melatonin supplementation in the management of periodontal disease. In this regard, some in vitro studies [22] and animal studies [23,24] have been performed and have demonstrated the beneficial effects of melatonin. In human studies, melatonin has been used either in the topical formulation (TF) or systemic formulation (SF) as a monotherapy or adjunctive therapy following SRP [6,7,8,25,26,27,28,29,30,31,32,33]. However, no systematic review and meta-analysis has critically appraised the use of melatonin in periodontitis. Hence, the present systematic review and meta-analysis were performed with the aim of qualitatively and quantitatively analyzing the published literature that has assessed melatonin in TF/SF for the management of periodontitis. To address this aim, the PICOS (population, intervention, comparison, outcome and study) criteria were set based on recently published literature [34] and are outlined in Table 1.

## 2. Materials and Methods

### 2.1. Protocol and Registration

The International Prospective Register of Systematic Reviews (PROSPERO) was thoroughly scrutinized for similar systematic reviews. No registered protocol could be found by reviewing the effects of melatonin TF/SF for the management of periodontitis. Thus, the protocol of the present review was formally registered with the PROSPERO database (registration number: CRD42020204144). The report was formulated according to the recommendations of the Preferred Reporting Items for Systematic Reviews and Meta-Analyses statement [35,36].

### 2.2. Inclusion Criteria

Interventional non-randomized clinical trials and randomized clinical trials (RCT) that have assessed melatonin TF/SF for the management of periodontitis in human subjects were included.

### 2.3. Exclusion Criteria

Letters to the editor, reviews, conference abstracts, in vitro studies, animal studies, and articles from which significant data could not be elucidated regarding the effects of melatonin on periodontal parameters were excluded.

### 2.4. Focus Question

This systematic review addressed two potential focus questions based on the PICOS criteria:

1. PICOS question 1: Is TF effective as a therapeutic measure in the management of periodontitis in human patients who have not undergone SRP?
2. PICOS question 2: Is SF effective as an adjunctive therapeutic measure in the management of periodontitis in human patients who have undergone SRP?

### 2.5. Search Strategy

The MesH terms “melatonin” AND “periodontitis” were searched in the Web of Science, PubMed, and Scopus as of November 2020. The identified articles were manually cross-referenced for further potential articles. Language filter was applied and only articles in English language were mined for data extraction and analysis. Articles published between 2000 and 2020 were chosen for the study and further subjected to application of the inclusion and exclusion criteria.

### 2.6. Study Selection and Data Extraction

(1) The identified articles were screened using titles and abstracts for potential duplicates and relevance to the topic of the present systematic review. Articles unrelated to the topic of the present review were excluded, along with duplicates.

(2) The full text of the screened articles was assessed using the selection parameters. Only articles that satisfied the inclusion criteria were part of the analysis.

Steps 1 and 2 were performed independently by two reviewers (TMB—Thodur Madapusi Balaji and SV—Saranya Varadarajan). The kappa coefficient (κ) was calculated to measure the reliability between the two reviewers and was found to be 0.97 and 0.98 for the reviewers TMB and SV, respectively. Significant data about the study characteristics (first author’s name, year of publication, country of origin), study design, sample size, melatonin/placebo formulation details, method and frequency of melatonin application, samples collected, markers measured, results, and inference were extracted from each of the included studies.

### 2.7. Risk of Bias Assessment, SIGN 50, and GRADE Scoring of the Included Studies

Risk of Bias Assessment tool (RoBANS) [37] and the Cochrane Collaboration Risk of Bias (RoB) tool [38] were used to assess the risk of bias for the included non-randomized and RCTs, respectively.

The SIGN 50 scoring criteria were used to assess the quality of the RCTs in the present systematic review [39].

A modification of the grading of recommendation assessment, development, and evaluation (GRADE) was employed [40,41] to assess the results of the included studies.

### 2.8. Meta-Analysis

The meta-analysis was carryout out using review manager (Rev Man) Version 5.4 (Cochrane, London, UK). The included studies were clearly assessed for homogeneity in clinical protocols and melatonin dosages used. The studies chosen after careful assessment were subjected to a meta-analysis performed using a statistical software and standardized protocol. The forest plot and statistical inferences that arose in the form of *p* value were evaluated for significance. Based on this a *p* value of <0.05 was considered significant while a *p* value of >0.05 was regarded as non-significant.

## 3. Results

### 3.1. Study Selection

**Figure 1:**
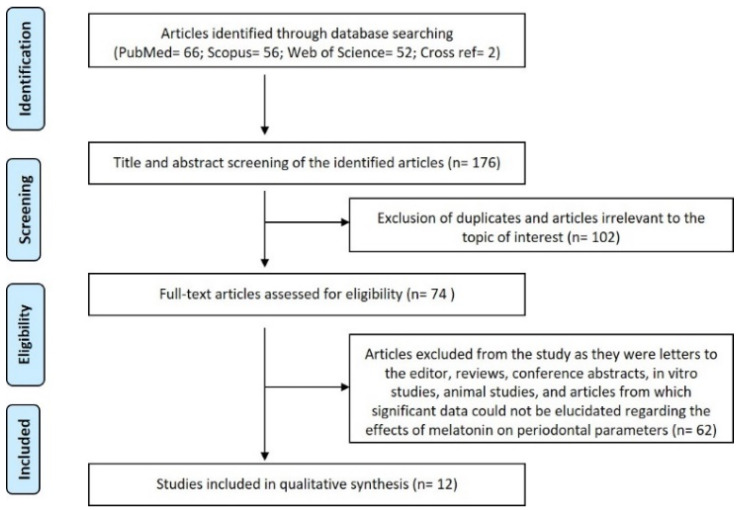
Summary of the search strategy employed in the systematic review.

A total of 176 articles (PubMed: 66, Scopus: 56, Web of Science: 52, and Cross reference: 2) were recognized. One hundred and two irrelevant/duplicate articles were excluded. Of the 74 articles scrutinized, only 12 articles satisfied the eligibility criteria and were recruited for qualitative analysis [6,7,8,25,26,28,29,30,31,32,33,42]. The kappa values for steps 1 and 2 of the review were 0.97 and 0.99, respectively. The selection strategy is depicted in the PRISMA flow chart (Preferred Reporting Items for Systematic Reviews and Meta-Analyses) (Figure 1).

### 3.2. Study Characteristics

All the studies included in the present systematic review were published between 2013 and 2020. Of the included studies, 4 were from Spain, 3 were from India, 3 were from Iran, and 1 each from Italy and Egypt. Six studies were clinical interventions without randomization, two were clinical interventional studies [6,25], two were clinical trials [8,26], and two were prospective longitudinal studies [30,32]. The other six studies were RCTs. Only four studies were randomized double-blinded placebo-controlled clinical trials [7,29,33,42], one study was a preliminary randomized triple-blind placebo-controlled clinical trial [31], and the other was a randomized single mask clinical trial [28]. One study did not administer a placebo [28], while another study did not mention placebo details [42]. The other four studies used placebo formulations [7,29,31,33] (All details available in Table 2 and Table 3 and Table S1, Table S2.).

Concerning melatonin, four studies used a TF denoted as Orabase cream with 1% melatonin [6,8,25,26]. The details of the studies included are presented in Table 2. All four studies prescribed melatonin/placebo cream for 20 days as a one-time night application without periodontal therapy. The effects were evaluated at baseline and 20 days after application.

Eight studies [7,28,29,30,31,32,33,42] used melatonin for oral consumption. The details of the studies included are presented in Table 3. Of these, one study used 1 mg melatonin [31], one study prescribed 2 mg melatonin [28], three studies used 3 mg melatonin [30,32,42], two studies used 6 mg melatonin [7,33], and one study prescribed 10 mg melatonin [29]. All studies advocated melatonin at night and subjected patients to SRP. One study recommended the use of chlorhexidine mouthwash (0.12%) [29], while another study recommended the use of chlorhexidine mouthwash (0.2%) until study completion [31]. Concerning the period of melatonin consumption, four studies prescribed melatonin for 4 weeks [28,30,32,42], two studies recommended the same for 8 weeks [7,33], one study advised the intake for 2 months [29], and the other recommended use for 1 month [31]. The follow-up appointments were heterogeneous. Three studies followed up patients at 0, 30, 60, and 90 days [30,32,42], two studies at 0, 90, and 180 days [28,29], two other studies at 0 and 8 weeks [7,33], and one study at 0 and 180 days [31]. One study compared melatonin 2 mg with melatonin 2 mg and vitamin C (60/75 mg) combination [28], and another compared melatonin with vitamin E (200 IU) as an adjunct to SRP [32]. All TF studies assessed melatonin as a topical application in patients with type 1 and 2 diabetes mellitus with periodontitis versus systemically and periodontally healthy subjects [6,8,25,26]. Amongst the SF studies, two evaluated melatonin on type 2 diabetic patients with severe periodontitis [7,33] while one study evaluated the potential of melatonin in patients with insomnia and periodontitis [29].

To assess treatment outcomes, the following were evaluated: pocket depth (PD), clinical attachment loss (CAL), gingival index (GI), periodontal disease index (PDI), community periodontal index (CPI), full-mouth plaque scores, full-mouth bleeding scores, and periodontitis-related biomarkers.

#### 3.2.1. Effect of Topical Melatonin Administration on Periodontal Parameters and Biomarker Levels

All interventional studies were not randomized. Out of the four studies recruited, three were performed on the same patient groups together at the same time point, and the results have been published in three different journals [6,8,25]. It was found that TF 1% Orabase cream application for 20 days could significantly reduce GI and PD in diabetic patients with periodontitis. Concerning the healthy subjects receiving placebo, data are unavailable. The first study reported a reduction in salivary osteopontin, osteocalcin, acid phosphatase, and alkaline phosphatase in the melatonin test group [25]. The second study reported a significant reduction in salivary RANK L and an increase in OPG (osteoprotegerin) levels following the intervention [6]. The third study found a reduction in salivary levels of CRP (serum C-reactive protein) and IL-6 (significant) and TNF-alpha (non-significant) [8]. The fourth study was also performed on the same patients included in the other three studies. However, it comprised an extra patient group. It was found that TF significantly reported a reduction in GI and PD in diabetic patients with periodontitis. There was also a reduction in GCF levels of IL-1 beta, IL-6, and PGE2 in the test group compared to the placebo group [26]. The details of the systemically and periodontally healthy subjects receiving placebo have not been provided.

#### 3.2.2. Effect of Systemic Melatonin Administration on Periodontal Parameters and Biomarker Levels

Out of the eight studies assessing systemic melatonin formulation [7,28,29,30,31,32,33,42], two were reported as prospective longitudinal studies, although they can be regarded as interventional studies without randomization [30,32]. One of these assessed 3 mg of melatonin administered for 4 weeks as an adjunct to SRP in chronic periodontitis patients versus patients treated by SRP only [30]. The study revealed a significant reduction in total leukocyte counts, neutrophils, and lymphocytes in the blood of patients in the melatonin group in 90 days. Another study evaluated a 4-week 3-mg melatonin use versus vitamin E 200 IU and no supplementation as an adjunct to SRP on plasma levels of vitamin C in chronic periodontitis patients [32]. The study found significantly elevated levels of vitamin C in plasma samples of periodontitis patients who received melatonin in contrast to vitamin E/no supplementation. The other six studies assessing systemic melatonin formulation were RCTs [7,28,29,31,33,42]. Two of these six studies evaluated the effects of melatonin supplementation on periodontal parameters only in periodontitis patients who underwent non-surgical periodontal therapy (NSPT). One study evaluated a 4-week 3-mg melatonin administration versus placebo on periodontitis patients who underwent SRP and found a significant reduction in GI, PDI, and CPI at 30-, 60-, and 90-days post-intervention in the test group [42].

The other study assessed the effects of 1 mg melatonin administered for 1 month versus placebo on periodontal parameters in periodontitis patients who underwent NSPT [31]. This study found a reduction in PD in both groups at 6 months following baseline. However, the melatonin group demonstrated a significant reduction in the 4–5 mm and >6 mm pockets in the test group compared to the placebo group.

Another study evaluating only periodontal parameters compared to a 4-week dose of 2 mg melatonin versus a combination of 2 mg melatonin and 60/75 mg of vitamin C and no placebo in patients who underwent NSPT [28]. This study found a significant reduction in GI, PD, and CAL in patients receiving a synergistic combination of melatonin and vitamin C compared to melatonin alone.

Three of these six studies evaluated biomarkers in addition to periodontal parameters following systemic melatonin administration [7,29,33]. One of the studies evaluated the effects of 2 monthly doses of 10 mg melatonin versus placebo on periodontal parameters and salivary biomarker levels and found a reduction in plaque index, GI, bleeding on probing, PD, CAL, along with a reduction in salivary TNF alpha levels and an overall improvement in insomnia [29]. The other study evaluated an 8-week dose of 6 mg melatonin versus placebo in type 2 diabetes mellitus patients with severe periodontitis and found a reduction in PD, CAL, and serum levels of IL-6 and CRP in the test group along with an increase in serum melatonin levels [7]. Another study by the same group with similar melatonin dose details and on the same patient groups demonstrated a reduction in IL-1 beta, malondialdehyde, and a concomitant increase in superoxide dismutase, glutathione peroxidase, catalase, and total antioxidant capacity in the test group versus the placebo group at 8 weeks post-intervention [33].

### 3.3. Risk of Bias Assessment

RoBANS [37] assessment revealed that out of the total 6 non-randomized interventional studies [6,8,25,26,30,32], four studies demonstrated a high risk of bias in three categories/domains, while two studies demonstrated a high risk of bias in four categories/domains, which tips in favor of the overall high risk of bias [30,32]. The data are presented in supplementary Table S3.

Based on the RoB tool [38], one study demonstrated a high risk of bias in four categories/domains [28] while the other 5 studies demonstrated a high risk of bias in only 1 category [7,29,31,33,42]. Despite this, many studies demonstrated unclear data. Hence, the overall risk of bias could still be regarded as high. The data are presented in supplementary Table S4.

### 3.4. SIGN 50 Scorings of RCT

The application of this scoring system tool to the RCT revealed the quality of the clinical trials. While 2 studies procured a minor score of [28,42], four studies procured a + score [7,29,31,33]. The data is presented in supplementary Table S5.

### 3.5. GRADE Scoring of the Recruited Studies

The GRADE evaluation revealed an overall weak recommendation in favor of all 12 studies assessing the effects of topical/systemic melatonin formulation [6,7,8,25,26,28,29,30,31,32,33,42]. The uniform weak score was attributed to the fact that none of the studies have reported a magnitude of the estimate of effect. Only one study reported side effects such as headache, nausea, and constipation in two patients. Although the GRADE score was low, it favored the use of melatonin for the management of periodontitis. A summary of the GRADE scoring is presented in supplementary Table S6.

### 3.6. Meta-Analysis

**Figure 2:**
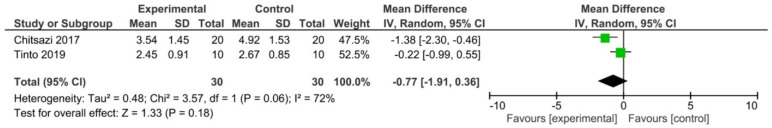
Meta-analysis summarizing the difference in pocket depth between the control (NSPT) and the experimental group (systemic melatonin application with NSPT).

Of the 12 recruited studies, a wide discrepancy was found concerning the dosage of melatonin, duration of administration, and period of evaluation. Only two studies out of the 12 studies had comparable data concerning PD and were subjected to a meta-analysis [28,31]. The meta-analysis was conducted only from a mathematical viewpoint as small sample meta-analysis are possible to perform. The meta-analysis revealed an overall trend in reduction PD in periodontitis patients receiving melatonin, although the overall difference was not statistically significant (Figure 2). However, the data from the meta-analysis cannot be interpreted or taken into account due to the low sample size and lack of any statistical inferences.

The rest of the periodontal parameters were not quantitatively analyzed as the studies had significant variation among key parameters.

## 4. Discussion

The present systematic review was done with the pursuit of critically appraising the studies concerning the administration of topical/systemic melatonin formulation for the management of periodontitis [6,7,8,25,26,28,29,30,31,32,33,42]. Of all the included studies, four were based on TF [6,8,25,26] while eight studies were based on SF [7,28,29,30,31,32,33,42]. All studies used melatonin to manage periodontitis, which is an irreversible periodontal condition characterized by gingival inflammation and bleeding, periodontal pockets associated with underlying alveolar bone destruction, and loss of attachment. Periodontitis is predominantly initiated by pathogenic bacteria, such as Porphyromonas gingivalis, Tannerrella forsythia, and Treponema denticola [43]. These organisms colonize the dental plaque biofilm and use it as an ecological niche for their survival. The toxins elaborated by the dental plaque pathogens can cause a microbial challenge to the host periodontal tissues, thereby causing inflammation and eliciting a chronic immune response characterized by infiltration of neutrophils, monocytes, and lymphocytes [44] accompanied by overproduction of pro-inflammatory cytokines and mediators such as prostaglandins, leukotrienes, and acute phase reactants such as C-reactive protein [45]. If inflammation could be regarded as a key mechanism in the pathobiology of periodontitis, another important mechanism that deserves attention is the role of oxidative stress and jeopardized antioxidant defense. It has been proven that periodontitis is a free radical (FR) disorder characterized by the overexpression of reactive oxygen species (ROS) [46]. Hence, the treatment of periodontitis revolves around debridement of the plaque biofilm and calculus that accumulate around the dentition, which is regarded as the cornerstone of periodontal therapy. It is not sufficient if only the dentition is attended to. It is also pivotal to perform debridement and instill plaque control measures around dental restorations and their interface with periodontal hard and soft tissues in order to prevent the initiation and progression of periodontitis. Additionally, therapeutic strategies include the administration of antimicrobials [2], anti-inflammatory agents [4], and antioxidants [3] systemically and topically for the management of periodontitis. It is in this connection that melatonin has been tried in periodontitis management.

Melatonin has been regarded as a multifaceted molecule. In the oral cavity, melatonin is synthesized by salivary glands [12] and gingival tissues [11], and it exerts antioxidant effects on the hydroxyl [47,48,49] peroxyl radicals, nitric oxide [50], and singlet oxygen molecules [51]. It should also be noted that melatonin accumulates in the mitochondria and augments mitochondrial metabolism [14]. Melatonin and its metabolites are generated after an oxidative encounter, such as AMK (N1-acetyl-5-methoxykynuramine) and AFMK, (Acetyl-N-formyl-5-methoxykynuramine) and are also equally potent FR scavengers [52]. It is also to be noted that melatonin stimulates the transcription and translation of several antioxidant enzymes [53]. Despite the short half-life of melatonin [54], it is still regarded as a natural antioxidant gift to living organisms.

The anti-inflammatory and immunomodulatory actions of melatonin have also been described in several in vitro and in vivo studies. It has been demonstrated that the pineal gland can crosstalk with the immune system [55]. It has been shown that melatonin is a double-edged sword concerning immune mechanisms [56]. It demonstrates pro-inflammatory and immunostimulatory effects by increasing neutrophil recruitment into inflammatory sites and also increases chemotaxis and neutrophilic phagocytosis [56]. Melatonin is a potent inhibitor of NF kappa B transcription, thereby reducing the production of IL-1 beta and TNF alpha [57]. Melatonin has been regarded as an anti-TNF alpha compound [58] and has been found to enhance antigen presentation by macrophages to T lymphocytes [59] that enhances the natural killer cell activity [60].

Studies on melatonin levels in periodontitis have reported significantly lower levels in saliva [19], gingival tissue samples [18], and GCF [20]. However, a recent systematic review highlighted a significantly low level of salivary melatonin in patients with periodontitis versus healthy individuals [21]. Since melatonin levels are lowered in periodontitis, an augmentation of the same by exogenous supplementation has been implemented in recent years.

In the present systematic review, four studies on TF [6,8,25,26] found effective results. It is to be reiterated at this point as earlier mentioned that three of these studies were performed on same patients [6,8,25] while the fourth study included an additional group [26]. However, there was a significant improvement in PD and GI values. This is an important finding, as in this situation, melatonin has been prescribed without plaque and calculus removal but has still been found to produce positive results. This could be justified by the fact that melatonin has high tissue penetrability property [61] and is a highly lipophilic molecule [62]. Moreover, it is highly bioavailable when topically applied. Concerning the change in GCF levels of markers, the above studies found a reduction in pro-inflammatory markers such as IL 1 beta, TNF-α, IL 6, and CRP [6,8,25,26]. This is plausible as the crevicular fluid subtly reflects the changes in the subjacent gingival tissues. A reduction in inflammatory burden in the gingival tissues and periodontium can produce changes in the marker profile in the GCF. Additionally, salivary levels of markers related to bone destruction, such as osteocalcin, osteopontin, acid phosphatase, alkaline phosphatase, RANK-L, and OPG underwent drastic changes [6,25]. There was a decrease in osteocalcin, osteopontin, acid phosphatase, alkaline phosphatase, and RANK L, and an increase in OPG levels in saliva following melatonin application. This is a point that needs elaboration as melatonin is regarded as a bone sparing agent and can significantly prevent bone resorption and increase bone anabolism [63]. The above changes reflect melatonin’s osteo-promotive properties. 

All eight studies prescribed the systemic melatonin formulations for oral consumption performed SRP in patients before supplementation [7,28,29,30,31,32,33,42]. In these studies, melatonin was used as an adjunctive agent in the management of periodontitis. In six of eight studies, periodontal parameters were measured [7,28,29,31,33,42], while two studies only measured the systemic levels of leukocytes and vitamin C after melatonin supplementation [30,32]. All six studies revealed that melatonin reduced PD, GI, PDI scores, gingival bleeding scores, and improved CAL gains. It was also remarkable that melatonin administration had a significant effect on the resolution of deep periodontal pockets >5 mm. These positive effects can be explained by the fact that melatonin administered systemically in these patients would reach the periodontium and gingival tissues to exert anti-inflammatory, antioxidant, and immunomodulatory effects [18].

This is possible because the GCF, which is a defense fluid of the periodontium, is an exudate of plasma and carries in it several molecules derived from systemic circulation [64]. Another possibility is through the gingival tissues, which are highly vascularized and oxygenated. In this situation, melatonin would have carried the systemic circulation to the gingival tissues and would have attained high concentrations therein. It could be argued that melatonin was only an adjunctive agent in these cases as SRP was performed and the changes in periodontal parameters could result from periodontal non-surgical therapy. However, this can be substantiated as there was a control/placebo group in all these studies compared to the melatonin group that had better periodontal changes. Concerning levels of inflammatory markers and oxidative stress markers, melatonin administration could produce a significant reduction in salivary levels of TNF alpha and serum levels of IL-6, CRP, IL 1 beta, and malondialdehyde, and a significant increase in the levels of superoxide dismutase, glutathione peroxidase, catalase, and total antioxidant capacity in addition to vitamin C levels in the blood [7,28,29,31,32,33,42]. Additionally, melatonin administration in periodontitis patients could also change the leukocyte profile and differential white blood cell counts and cause a reduction in neutrophil and lymphocyte counts [30]. All the above data only reveal the protective properties of melatonin in periodontal homeostasis. One of the eight studies also found melatonin in periodontitis patients with insomnia to cure both conditions [29]. In the above-mentioned study, there was a significant improvement in periodontal parameters, reduction in salivary TNF alpha levels, and restoration of sleep pattern, as detected by the Athens insomnia scores. This is plausible because melatonin is a principal sleep-promoting molecule that restores the biological clock in addition to its numerous other functions in the human system.

One of the studies assessed that melatonin with vitamin C combination found superior effects on periodontal parameters versus only melatonin administration [28]. This is an interesting finding, as both melatonin and vitamin C are potent antioxidants and the synergism between the two would have produced additional effects. Another study in the systemic review compared melatonin with vitamin E administration and found that the periodontal benefits of melatonin were far superior compared to vitamin E [32]. This can be justified as melatonin is superior compared to conventional antioxidants.

Within the given scope, the present systematic review highlights significant information about the use of melatonin as a therapeutic agent in periodontitis management. However this review also sheds light on several limitations that are inherent to the included studies listed hereafter. The data from the 12 studies included in this review should be interpreted with caution as melatonin doses were ranging from 1–10 mg used in the studies. The dosage was administered in different periods, and clinical assessment was also performed at different times. An RoB assessment revealed that all 12 studies had a high risk of bias. The SIGN 50 scoring criteria applied to the RCT revealed that two trials obtained a − score and four trials obtained only a + score. The GRADE assessment also revealed an overall weak recommendation for all the 12 included studies, however, in favor of using melatonin for managing periodontitis. This outcome arose because melatonin did not produce adverse effects in most of the studies, except one where nausea and vomiting were noted in two patients [29]. Moreover, a meta-analysis could be performed only by recruiting two studies [28,31]. This was because of data heterogeneity. However, the meta-analysis was still performed despite numerous inherent disadvantages since it was possible to perform the same from a mathematical viewpoint. The results of the meta-analysis revealed that melatonin had a positive influence (although non-significant) on the reduction of PD in periodontitis patients receiving 1–2 mg melatonin for oral consumption in addition to SRP. This inference cannot be considered vital as the sample size of the meta-analysis was low and moreover, statistical significance was not obtained. Hence it would be prudent on the readers part to understand that the findings of this systematic review emanate from the review protocol per se rather than the meta-analysis findings which can be read only as a part of the protocol and cannot be used for any conclusive evidence.

Based on the assessed literature, melatonin can be considered as a potential host modulatory agent in periodontal therapy due to its beneficial properties, good safety profile, and minimal side effects. However, data from this systematic review should be cautiously interpreted due to the associated high risk of bias. Only well-planned RCTs with adequate blinding that conform to the CONSORT (Consolidated Standards of Reporting Trials) guidelines should be encouraged.

It is to be reiterated that SRP should be performed before melatonin administration as it is a prerequisite for obtaining tangible periodontal benefits. Further studies should be performed after dose titration, frequency, and therapy time standardization to exploit the fullest benefits of melatonin as an adjunctive agent in periodontal therapy. Additionally, it would be worthwhile to try other formulations containing melatonin such as mouthwashes, gummies, lozenges, and targeted local drug delivery preparations containing melatonin coated microspheres and nanospheres for management of periodontitis. 

## 5. Conclusions

The results of the present systematic review allow to cautiously suggest that melatonin as a topical and systemic formulation could be a potential host modulatory agent for periodontitis management. However, the available data should be interpreted carefully due to the associated high risk of bias. Moreover, dose titration, frequency, and formulation type need to be standardized and further well designed RCTs should be carried out to obtain more solid clinical conclusions and indications.

## Figures and Tables

**Table 1:** PICOS criteria for the recruited studies.

PICOS Criteria Category	Details of the Recruited Studies
population	Patients diagnosed with clinical features of periodontitis and periodontally and systemically healthy individuals
intervention	Melatonin in topical formulation as monotherapy for management of periodontitis Melatonin in systemic formulation as adjunctive therapy following scaling and root planing
comparison	Periodontal and biochemical parameters in the melatonin versus the placebo/group receiving no treatment
outcomes	Pocket depth, clinical attachment loss, gingival index, periodontal disease index, community periodontal index, salivary and plasma levels of cytokines, oxidant and antioxidant markers
study	Non randomized interventional studies Randomized controlled clinical trials

**Table 2:** Detailed data extracted from the included studies using topic melatonin formulation.

S.No	First Author Name/Year of Publication/Country of Origin	Type of Study/Sample Size	Melatonin/ Placebo Formulation Details	Method and Frequency of Melatonin Application with Details of the Intervention	Samples Collected	Markers Measured	Results	Inference
1.	Antonio Cutando/2013/Spain	Clinical intervention study with 2 groups Healthy subjects (30): placebo group Diabetic patients with periodontal disease (30): Test group	Test group: orabase cream with 1% melatonin Placebo group: ora base cream without melatonin	No periodontal treatment performed. All subjects in the study were instructed to apply orabase formulation (the amount that fit toothbrush surface) on upper and lower arch attached gingiva surfaces with aid of toothbrush in the night time for 20 days	Fasting stimulated saliva and plasma samples collected at baseline and 20 days post-application	Salivary acid phosphatase (ACP) and alkaline phosphatase (ALP) measured by spectrophotometric method. Salivary osteocalcin (OCN) measured by electrochemiluminescence method. Salivary osteopontin (OPN) measured by sandwich ELISA technique.	Values provided only for group 2: Baseline findings: Mean Gingival index: 15.8 ± 10.26 Mean Pocket depth: 28.3 ± 19.48 Mean ALP: 40.51 ± 4.83 Mean ACP: 83.08 ± 6.85 Mean OCN: 5.83 ± 1.41 Mean OPN: 12.49 ± 1.78 After 20 days of intervention: Mean Gingival index: 5.59 ± 4.08 Mean Pocket depth: 11.9 ± 9.01 Mean ALP: 26.88 ± 4.03 Mean ACP: 43.2 ± 5.52 Mean OCP: 5.78 ± 1.39 Mean OPN: 8.34 ± 1.45 The pocket depth and gingival index reduced significantly following melatonin application and correlated with all 4 markers significantly.	Melatonin application reduced the gingival index and pocket depths significantly and also, the levels of the 4 markers measured changes in gingival index correlated with salivary acid phosphatase and osteopontin while pocket depth changes correlated with acid, alkaline phosphatase, and osteopontin
2.	Antonio Cutando/2014/Spain	Clinical intervention study with 2 groups Healthy subjects (30): placebo group. Diabetic patients with periodontal disease(30): test group	Test group: orabase cream with 1% melatonin. Placebo group: ora base cream without melatonin	No periodontal treatment performed. All subjects in the study were instructed to apply orabase formulation (the amount that fit toothbrush surface) on upper and lower arch attached gingiva surfaces with aid of toothbrush in the night time for 20 days	Fasting stimulated saliva samples collected at baseline and 20 days post-application	Salivary and plasma melatonin(radioimmunoassay) measured only at baseline. Salivary Receptor activator of nuclear factor kappa B ligand (RANKL) and osteoprotegerin (OPG) (ELISA) measured at baseline and 20 days after clinical intervention	Values provided only for group 2: Baseline findings: Mean Gingival index: 15.8 ± 10.26 Mean Pocket depth: 28.3 ± 19.48 Mean salivary RANKL: 102.6 ± 66.67 Mean salivary OPG: 10.4 ± 7.61 After 20 days of intervention: Mean Gingival index: 5.6 ± 4.08 Mean Pocket depth: 11.9 ± 9.01 Mean salivary RANKL: 73.5 ± 47.39 Mean salivary OPG: 16.9 ± 7.20 Significant reduction in pocket depth, gingival index, and salivary RANK L levels and a significant increase in salivary OPG values were observed following intervention (*p* < 0.001). the decrease in pocket depth and gingival index correlated with a parallel increase in salivary OPG values.	Melatonin is a protective molecule in periodontal treatment especially in diabetic patients. Melatonin application reduced the gingival index and pocket depths significantly Melatonin application could inhibit RANK L mediated bone resorption in could alter the RANKL OPG ratios favorably.
3.	Antonio Cutando/2015/Spain	Clinical trial study with 2 groups Healthy subjects (30): placebo group. Diabetic patients with periodontal disease (30): test group	Test group: orabase cream with 1% melatonin, 16.5% sodium carboxymethyl cellulose, 16.5% pectin, 16.5% jello, plastibase c.s.p 100. Placebo group: ora base cream with the same composition as above without melatonin	No periodontal treatment performed. All subjects in the study were instructed to apply orabase formulation (the amount that fit toothbrush surface) on upper and lower arch attached gingiva surfaces with aid of toothbrush in the night time for 20 days	Fasting stimulated saliva and plasma samples collected at baseline and 20 days post-application	Salivary and plasma melatonin(radioimmunoassay) measured only at baseline. Serum C-reactive protein (CRP) measured by nephelometry and TNF alpha and IL 6 measured by ELISA	Values provided only for group 2: Baseline findings: Gingival index: 15.84 ± 10.26 Pocket depth: 28.29 ± 19.48 TNF alpha: 1.79 ± 0.19 IL 6: 0.57 ± 0.07 CRP: 0.39 ± 0.11 After 20 days of intervention: Gingival index: 5.59 ± 4.08 Pocket depth: 11.90 ± 9.01 TNF alpha: 1.76 ± 0.19 IL 6: 0.47 ± 0.07 CRP: 0.31 ± 0.11 All markers measured correlated positively with gingival index and pocket depth values.	Melatonin topical application for 20 days resulted in a significant reduction of pocket depth, gingival index, IL 6, and CRP values (*p* < 0.001) but not that of TNF alpha (*p* > 0.001) There was a positive correlation of all the 3 markers with gingival index and pocket depth after the intervention which meant that a reduction of pocket depth and gingival index also denoted a significant parallel reduction in the level of the markers It was concluded that melatonin application modulates the inflammatory response in periodontal disease
4.	Javier Montero/2017/Spain	Clinical trial study with 3 groups DMPaMT: Diabetic patients with periodontal disease who were administered melatonin (30) DMP-Placebo: Diabetic patients with periodontal disease who were administered placebo (30) NoDM: systemically and periodontally healthy individuals who were administered placebo (30)	Test group: orabase cream with 1% melatonin. Placebo group: ora base cream without melatonin	No periodontal treatment performed. All subjects in the study were instructed to apply orabase formulation (the amount that fit toothbrush surface) on upper and lower arch attached gingiva surfaces with aid of toothbrush in the night time for 20 days	Gingival crevicular fluid (GCF) was sampled from 2 periodontal pocket sites and pooled for the assays	GCF IL 1 beta, IL 6, and PGE 2 were measured by ELISA	Values provided for group 1 and 2 only at baseline and after 20 days of orabase application: Baseline findings: Group 1: Gingival index: 15.84 ± 10.2 Pocket depth: 2.8 ± 1.9 GCF IL 1 beta: 127.73 ± 99.50 IL 6: 0.57 ± 0.07 PGE2: 265.42 ± 101.60 Group 2: Gingival index: 14.51 ± 9.70 Pocket depth: 2.7 ± 1.5 GCF IL 1 beta: 122.47 ± 95.2 IL 6: 0.56 ± 0.07 PGE2: 263.45 ± 98.7 After 20 days of intervention: Group 1: Gingival index: 5.59 ± 4.08 Pocket depth: 1.8 ± 1.2 GCF IL 1 beta: 114.34 ± 74.88 IL 6: 0.47 ± 0.07 PGE2: 222.78 ± 87.88 Group 2: Gingival index: 14.13 ± 10.15 Pocket depth: 2.6 ± 1.2 GCF IL 1 beta: 120.93 ± 101.4 IL 6: 0.54 ± 0.07 PGE2: 260.26 ± 99.1 All values were statistically significant in Group 1 following the intervention. No significance was found in parameters before and after intervention in group 2. There was a direct and significant correlation between GCF IL1 beta, IL 6 and PGE2 values and gingival index and pocket depth in Group 1 while this correlation was absent in Group 2	Treatment of diabetic patients with periodontal disease with topical melatonin improved the gingival index and pocket depth significantly and resulted in a significant reduction in the levels of pro-inflammatory mediators IL 1 beta, IL 6 and PGE2

**Table 3:** Detailed data extracted from the included studies using systemic melatonin formulation.

S.No	First Author Name/Year of Publication/Country of Origin	Type of Study/Sample Size	Melatonin/Placebo Formulation Details	Method and Frequency of Melatonin Application with Details of the Intervention	Samples Collected	Markers Measured	Results	Inference
1.	Marawar A.P/2014/India	A randomized, double-blind, placebo-controlled, comparative clinical study with 2 groups Group A(80): Periodontitis patients receiving placebo after scaling and root planing Group B(80): Periodontitis patients receiving melatonin after scaling and root planing	Melatonin 3 mg tablet formulation for oral consumption Placebo details not mentioned	Melatonin tablet of 3 mg dosage was prescribed in the test group (Group B) as a daily night once dose for 4 weeks following scaling and root planing Placebo was prescribed in Group A in the same manner and frequency as above	No samples were collected	No molecular or biochemical markers measured	Gingival index Group A Baseline: 1.86 ± 0.60 30 days: 1.73 ± 0.59 60 days: 1.65 ± 0.60 90 days: 1.56 ± 0.58 Group B Baseline:1.77 ± 0.55 30 days: 1.52 ± 0.59 60 days: 1.24 ± 0.55 90 days: 1.01 ± 0.56 Statistically significant lowering of Gingival index found in the test group at 30, 60, 90 days compared to the placebo group. Periodontal disease index Group A Baseline: 4.73 ± 0.52 30 days: 4.71 ± 0.50 60 days: 4.53 ± 0.49 90 days: 4.49 ± 0.49 Group B Baseline: 4.67 ± 0.43 30 days: 4.51 ± 0.48 60 days: 4.49 ± 0.34 90 days: 4.27 ± 0.33 Statistically significant lowering of Periodontal disease index found in the test group at 30, 90 days compared to the placebo group. Community periodontal index Group A Baseline: 4.69±0.95 30 days: 4.56 ± 0.98 60 days: 4.46 ± 0.94 90 days: 4.41 ± 0.91 Group B Baseline: 4.51 ± 1.06 30 days: 2.96 ± 1.03 60 days: 2.68 ± 0.98 90 days: 2.41 ± 0.94 Statistically significant lowering of Community periodontal index found in the test group at 30, 60, 90 days compared to the placebo group (*p* < 0.05)	In the present study, the gingival index indicated gingival inflammation, the Periodontal disease index and Community periodontal index indicated connective tissue and bone loss. Melatonin administration was found to improve the gingival index, periodontal disease index, and community periodontal index values as an adjunctive agent following scaling and root planing. Melatonin has been inferred to combat oxidative stress and inflammation and regulates some components of the immune system thereby aiding in the management of periodontal disease
2.	Chitsazi M/2017/Iran	Randomized single mask clinical trial with 3 groups Group 1(20): Chronic periodontitis patients who underwent only non-surgical therapy Group 2(20) Chronic periodontitis patients who underwent non-surgical therapy along with melatonin Group 3(20): Chronic periodontitis patients who underwent non-surgical therapy along with melatonin and vitamin C	Melatonin tablet 2 mg tablet formulation for oral consumption with or without Vitamin C tablets (60 mg for female patients and 75 mg for male patients) No placebo was used in the present study	Group 1 received scaling and root planing with no adjunctive medication or placebo Group 2 received scaling and root planing followed by melatonin 2 mg once daily dose for 4 weeks Group 3 received scaling and root planing followed by melatonin 2 mg and Vitamin C(60/75 mg) once daily dose for 4 weeks	No samples collected	No molecular or biochemical markers measured	Gingival index(GI) difference values: Group 1: Baseline to 3 months: 0.45 ± 0.13 Baseline to 6 months: 0.63 ± 0.24 3 to 6 months: 0.33 ± 0.22 Group 2: Baseline to 3 months: 0.54 ± 0.12 Baseline to 6 months: 0.83 ± 0.21 3 to 6 months: 0.62 ± 0.14 Group 3: Baseline to 3 months: 0.63 ± 0.11 Baseline to 6 months: 0.92 ± 0.22 3 to 6 months: 0.75 ± 0.13 Probing depth(PD) in mm: Group 1: Baseline: 6.40 ± 1.20 3 months: 5.23 ± 1.89 6 months: 4.92 ± 1.53 Group 2: Baseline: 6.41 ± 1.02 3 months: 4.56 ± 1.31 6 months: 3.54 ± 1.45 Group 3: Baseline: 6.43 ± 1.17 3 months: 4.41 ± 1.15 6 months: 3.08 ± 1.12 Clinical attachment loss(CAL): Group 1: Baseline: 6.23 ± 1.22 3 months: 5.14 ± 1.23 6 months: 4.56 ± 1.16 Group 2: Baseline: 6.29 ± 1.16 3 months: 4.23 ± 1.43 6 months: 3.22 ± 1.52 Group 3: Baseline: 6.30 ± 1.21 3 months: 4.12 ± 1.95 6 months: 3.00 ± 1.53 Mean GI was significantly lower in group 2 and group 3 compared to baseline after 3 and 6 months (*p* < 0.001), however, the difference between 3 and the 6-month interval was not significant (*p* > 0.05) Scaling and root planing improved PD and CAL values at 3 months and 6 months in all the three groups compared to baseline (*p* < 0.001). Significant PD and CAL reduction was seen in Group 3 compared to Group 1 and 2 at 6 months compared to 3 months (*p* < 0.05). This change was not seen in Group 1 and 2 (*p* > 0.05)	Melatonin plays a critical role in the pathogenesis of the periodontal disease Vitamin C is known to play a critical role in matrix remodeling and connective tissue homeostasis in the periodontium. Previous studies on Vitamin C administration after non-surgical periodontal therapy have not shown significant improvement in periodontal parameters However, a combination of Melatonin and Vitamin C is according to the present study more efficient in combating periodontal disease as Vitamin C is known to recycle and replenish melatonin level
3.	Hadi Bazyar/2019/Iran	Randomized double-blinded placebo-controlled single-center trial with 2 groups The control group (22) Type 2 diabetes mellitus patients with severe symptoms of periodontal disease underwent non-surgical periodontal therapy (NSPT) including scaling and root planing with dental hygiene instructions followed by placebo tablet consumption for 8 weeks Intervention group (22) Type 2 diabetes mellitus patients with severe symptoms of periodontal disease underwent non-surgical periodontal therapy (NSPT)including scaling and root planing with dental hygiene instructions followed by melatonin tablet consumption for 8 weeks	Melatonin 250 mg tablets were procured from Nature Made, USA, composed of 3 mg melatonin net, sodium starch glycolate, magnesium stearate Placebo 250 mg tablets were made in Ahvaz Jundishapur University containing cellulose, silicon dioxide, magnesium stearate, and starch with peppermint oil for flavor	The Control group received NSPT followed by 2 placebo tablets once a day for 8 weeks to be consumed 1 h before bedtime. The intervention group received NSPT followed by 2 melatonin tablets once a day for 8 weeks to be consumed 1 h before bedtime.	Blood sample (5 mL) collected at baseline and 8 weeks post NSPT after 12 h overnight fasting	Serum melatonin, IL-6, TNF- alpha, and hc-CRP were measured in the samples using commercially available ELISA kits	Control group: Melatonin Baseline: 4.32 ± 1.93 pg/mL Post intervention: 4.07 ± 1.91 pg/mL TNF alpha Baseline: 8.65 ± 3.87 pg/mL Post intervention: 8.5 ± 3.95 pg/mL IL 6 Baseline: 2.16 ± 0.9 pg/mL Post intervention: 2.08 ± 0.87 pg/mL Hc CRP Baseline: 2.31 ± 0.96 pg/mL Post intervention: 2.4 ± 0.94 pg/mL Probing depth Baseline: 4.54 ± 1.01 mm Post-intervention: 4.36 ± 1.04 mm Clinical attachment loss Baseline: 3 ± 0.75 mm Post-intervention: 2.77 ± 0.68 mm Plaque Baseline: 22 Post-intervention: 18 Bleeding on probing Baseline: 22 Post intervention: 20 Intervention group: Melatonin Baseline: 4.52 ± 1.78 pg/mL Post intervention: 5.03 ± 1.68 pg/mL TNF alpha Baseline: 9.05 ± 3.56 pg/mL Post intervention: 8.24 ± 3.45 pg/mL IL 6 Baseline: 2 ± 0.92 pg/mL Post intervention: 1.42 ± 0.73 pg/mL Hc CRP Baseline: 2.53 ± 0.77 pg/mL Post intervention: 1.6 ± 0.91 pg/mL Probing depth Baseline: 4.45 ± 0.96 mm Post intervention: 2.59 ± 1.04 mm Clinical attachment loss Baseline: 3.04 ± 0.78 mm Post intervention: 1.59 ± 0.59 mm Plaque Baseline: 22 Post intervention: 13 Bleeding on probing Baseline: 22 Post intervention: 15 Melatonin levels in serum increased significantly in the intervention group post-treatment. Additionally, IL 6 and hs CRP levels were significantly decreased in the intervention group (*p* < 0.05). but not in the control group (*p* > 0.05) post for 8 weeks. Melatonin supplementation along with NSPT significantly lowered probing depth and clinical attachment loss values post 8 weeks compared to baseline (*p* < 0.05). in the control group, NSPT alone led to a significant lowering of clinical attachment values 8 weeks post-treatment (*p* < 0.05). In both, the groups no improvement in plaque and bleeding on probing values was noted post 8 weeks compared to baseline(*p* > 0.05)	Diabetes mellitus type 2 modulates and worsens periodontal disease and vice versa Melatonin supplementation improves the periodontal status of diabetes type 2 patients by altering the levels of IL-6 and hs CRP. No effect was exerted by melatonin supplementation of TNF alpha values in serum. It was noted that melatonin supplementation with non-surgical periodontal therapy also led to a significant lowering of pocket depth and clinical attachment loss Melatonin supplementation also significantly raised serum melatonin levels Melatonin supplementation may be used as a significant adjunct in periodontal management of type 2 diabetic subjects
4.	Hesham-El-Sharkawy/2019/Egypt	A randomized placebo-controlled clinical trial with 2 groups Melatonin plus SRP/Group 1(38): Chronic periodontitis patients with insomnia receiving melatonin supplementation following scaling and root planing Placebo plus SRP/Group 2(36): Chronic periodontitis patients with insomnia receiving placebo following scaling and root planing	10 mg oral melatonin capsule (Puritans pride, Inc., Holbrook, NY, USA) was given to the Group 1 participants An equivalent placebo was given to the Group 2 participants, composition not mentioned	Both groups received thorough scaling and root planing following periodontal diagnosis and inclusion into the study. Thorough oral hygiene instructions were given to the patients and 0.12% chlorhexidine mouthwash was prescribed for 2 weeks after scaling and root planing. The patients were also subjected to professional scaling and deplaquing every month until the trial was completed. 10 mg melatonin or placebo was given to the patients 1 h before bedtime as a daily dose for 2 months from the start of the trial	Saliva samples were collected from the patients after overnight fasting between 8 and 10 a.m. at baseline, 3 and 6 months after therapy. Whole unstimulated saliva was sampled by expectoration into 5 mL sterile polypropylene tubes and used for the study	Tumor necrosis factor-alpha was measured in the saliva samples using the ELISA technique	Group 1: PI: Baseline: 2.35 ± 0.45 3 months: 0.84 ± 0.26 6 months: 0.81 ± 0.23 GI: Baseline: 2.14 ± 0.36 3 months: 0.73 ± 0.19 6 months: 0.68 ± 0.17 BOP (%) Baseline: 63 ± 21 3 months: 11 ± 2.3 6 months: 12 ± 2.1 PD: Baseline: 4.3 ± 0.8 3 months: 2.4 ± 1.0 6 months: 2.3 ± 0.9 CAL: Baseline: 4.8 ± 0.9 3 months: 2.7 ± 1.1 6 months: 2.6 ± 1.0 Group 2: PI: Baseline: 2.44 ± 0.67 3 months: 0.92 ± 0.14 6 months: 0.95 ± 0.17 GI: Baseline: 2.21 ± 0.24 3 months: 0.67 ± 0.14 6 months: 0.69 ± 0.15 BOP (%) Baseline: 59 ± 19 3 months: 16 ± 2.2 6 months: 18 ± 2.8 PD: Baseline: 4.4 ± 0.7 3 months: 3.1 ± 0.9 6 months: 3.0 ± 0.8 CAL: Baseline: 4.7 ± 1.0 3 months: 3.5 ± 0.9 6 months: 3.4 ± 1.2 PD and CAL were significantly reduced in both group 1 and group 2 at 3 and 6 months compared to baseline (*p* < 0.001). However, a more effective reduction in PD and CAL were noted in Group 1 compared to group 2 (*p* < 0.01). the PI, GI, BOP values were also significantly lower in both the groups after 3 and 6 months compared to baseline (*p* < 0.05), with no variation between the 3 months and 6 months values (*p* > 0.05). The mean TNF alpha values in saliva samples and AIS scores have been only graphically depicted without actual numerical values. It was found that there was a statistically significant reduction in salivary TNF alpha and AIS scores in Group 1 compared to group 2 (*p* < 0.01). Correlation analysis found no significant correlation between salivary TNF alpha values with any of the other parameters assessed in both the groups	In the present trial, the efficacy of melatonin in the management of insomnia and periodontal disease was assessed. A combination of melatonin plus scaling and root planing was found to effectively reduce PD and improve CAL values and also resulted in a significant reduction of salivary TNF alpha and AIS (Athens Insomnia Score) scores. The study concluded that 10 mg of melatonin given once in the night as an adjunct to periodontal treatment will result in improvement of periodontal parameters and sleep pattern. This could also be a treatment modality in patients without sleep disorders. The effects of melatonin on the reduction of salivary TNF alpha and AIS shows its health benefits
5.	Marawar A.P/2019/India	Mentioned as a prospective longitudinal study with 2 groups: Group A(80): chronic periodontitis patients who underwent scaling and root planing alone Group B(80): chronic periodontitis patients who underwent scaling and root planing along with melatonin supplementation.	Melatonin 3 mg tablets (brand not mentioned), composition unknown No placebo tablets administered in the study.	Both the groups visited on day 0 termed baseline and were subjected to full-mouth scaling and root planing. Group A received no medication while group B patients were asked to take one 3 mg melatonin tablet at night for 4 week.	Blood samples were obtained from the patients for leukocyte studies at baseline, day 30, day 60, and day 90. The volume of blood and method of drawing blood sample not mentioned.	Total leukocyte count (TLC), Differential leukocyte count (DLC), and Erythrocyte sedimentation rate (ESR) were the markers studied in the blood samples.	TLC: Group A Baseline: 9117.5 ± 2103.9 Day 30: 9112.5 ± 2098.4 8 Day 60: 8886.2 ± 1783.5 Day 90: 8531.6 ± 1555.4 Group B Baseline: 9370 ± 2278.7 Day 30: 8916.2 ± 1868.1 Day 60: 9205.5 ± 1719.6 Day 90: 7593.7 ± 1493.2 Neutrophils%: Group A Baseline: 74.05 ± 6.15 Day 30: 73.87 ± 5.09 Day 60: 73.97 ± 5.55 Day 90: 75.18 ± 5.79 Group B Baseline: 74.71 ± 6.64 Day 30: 75.57 ± 5.24 Day 60: 74.29 ± 4.87 Day 90: 73.10 ± 4.17 Lymphocytes%: Group A Baseline: 22.70 ± 4.84 Day 30: 23.50 ± 4.89 Day 60: 22.78 ± 4.98 Day 90: 21.62 ± 5.36 Group B Baseline: 22.30 ± 5.88 Day 30: 22.30 ± 5.88 Day 60: 21.67 ± 4.59 Day 90: 22.86 ± 4.34 Eosinophils% Group A Baseline: 0.41 ± 0.84 Day 30: 0.16 ± 0.43 Day 60: 0.38 ± 1.58 Day 90: 0.22 ± 0.55 Group B Baseline: 0.31 ± 0.68 Day 30: 0.27 ± 0.74 Day 60: 0.27 ± 0.55 Day 90: 0.35 ± 0.71 Monocytes% Group A Baseline: 0.95 ± 1.04 Day 30: 0.68 ± 0.72 Day 60: 0.91 ± 0.84 Day 90: 1.0 ± 0.95 Group B Baseline: 0.82 ± 0.97 Day 30: 0.65 ± 0.85 Day 60: 0.63 ± 0.78 Day 90: 0.71 ± 0.79 Basophils%: Group A Baseline: 1.85 ± 1.57 Day 30: 1.67 ± 1.33 Day 60: 1.95 ± 1.70 Day 90: 1.87 ± 1.80 Group B Baseline: 1.64 ± 1.39 Day 30: 1.65 ± 1.64 Day 60: 1.80 ± 1.48 Day 90: 1.73 ± 1.35 A higher percentage of male patients participated in the study. A highly significant reduction in total leukocyte counts was noted at 90 days in the melatonin group (Group B) compared to Group A (*p* < 0.01). Additionally, a highly significant reduction in neutrophil and lymphocyte counts was noted in group B compared to group A (*p* < 0.01). No differences were observed in ESR values between the groups (numerical values not provided).	No human study done on patients with periodontal disease to assess the hematological changes brought about by melatonin Only one animal study has reported a lowering of granulocyte counts in cattle receiving melatonin therapy. The present study results also show a similar trend. Melatonin has been proven to be an efficient immunomodulator and can activate several components of the immune system to modulate the pathogenesis of the periodontal disease.
6.	Manuel Tinto/2020/Italy	A preliminary randomized triple-blind placebo-controlled study with 2 groups: Group 1 (control group) (10): periodontitis patients who underwent non-surgical periodontal therapy following one-stage full-mouth protocol associated with oral administration of placebo tablet 1 mg at bedtime for 30 days. Group 2 (experimental group) (10): periodontitis patients who underwent non-surgical periodontal therapy following one stage full mouth protocol associated with oral administration of melatonin tablet 1mg at bedtime for 30 days	Melatonin tablets 1 mg (Farmacia Parati–Dr.SSA Simona Corti, Lentate Sul Seveso, Italy) containing synthetic melatonin, pregelatinized starch USP 22, magnesium stearate, silicon dioxide. Placebo tablets 1mg (Farmacia Parati–Dr.SSA Simona Corti, Lentate Sul Seveso, Italy) containing pregelatinized starch USP 22, magnesium stearate, silicon dioxide.	Both the groups underwent non-surgical periodontal therapy (NSPT) under local anesthesia as a one-stage procedure using ultrasonic instruments and periodontal curettes. Time spent per quadrant was nearly 45 min. Patients were instructed to follow strict oral hygiene and plaque control and were instructed to use 0.2% chlorhexidine mouthwash (Corsodyl mouthwash, GlaxoSmithKline consumer Healthcare S.p. A, Verona, Italy). One anonymous blister pack containing melatonin/placebo tablets were given to the patient to be consumed once a day at bedtime for 30 days	No samples were collected from the patients	No biochemical or molecular markers assessed	Group 1 PD at baseline: 3.40 ± 0.83 PD at 6 months: 2.67 ± 0.85 Group 2 PD at baseline: 3.72 ± 0.90 PD at 6 months: 2.45 ± 0.91 Results of primary outcome: Mean PD change (standard deviation value) of teeth with pockets 4–5 mm and >6 mm: Group 1: PD 4–5 mm: 1.04 (0.69) PD > 6 mm: 2.11 (0.96) Group 2: PD 4–5 mm: 1.86 (0.81) PD > 6 mm: 3.33 (1.43) Both the groups exhibited an overall change in PD at 6 months compared to baseline (*p* < 0.05). However, no differences were observed between melatonin and placebo group Concerning FMBS% (Full-Mouth Bleeding Score), FMPS% (Full-Mouth Plaque Score)scores no differences were observed between the 2 groups although numerical values are not mentioned Concerning the primary outcome measured, that is the PD change at 6 months compared to baseline for the subgroups, it was found that melatonin was more efficient than placebo in reducing probing depth in both 4–5 mm and >6 mm pockets (*p* < 0.0001)	Melatonin is considered an efficient host modulatory agent and was well tolerated by all the participants in the present study. The long term effect of melatonin on reducing probing depth is linked to its pleiotropic functions on the immune and antioxidant systems. In the present study with its limitations of low sample size and a low dose of melatonin (1 mg) as recommended by the Italian Ministry of health, melatonin administration was found to provide non-pharmacological support in periodontal healing after non-surgical periodontal therapy.
7.	Marwar A.P/2020/India	Mentioned as a prospective longitudinal study with 3 groups Group A(80): patients with chronic periodontitis who underwent scaling and root planing alone Group B(80): patients with chronic periodontitis who underwent scaling and root planing followed by supplementation with Vitamin E 200 IU at night for 4 weeks Group C(80): patients with chronic periodontitis who underwent scaling and root planing supplemented with melatonin tablet 3 mg daily at night for 4 weeks	Melatonin 3 mg tablets (no manufacturers name or composition mentioned Vitamin E 200 IU (no manufacturers name or composition mentioned No placebo formulation used in the present study.	Participants visited on day 0 and were screened and allocated to one of the 3 groups. No randomization was followed. No blinding details were provided. The patients underwent scaling and root planing. Details of technique not elaborated. They were subjected to either melatonin/Vitamin E tablets daily night for 4 weeks. Recall visits were scheduled on day 30, 60 and 90	Blood samples were collected from the patients. Detailed protocol not mentioned	Vitamin C assay was done in the blood samples obtained at baseline, day 30, day 60, and day 90	Antioxidant Vitamin C levels in blood samples: Baseline: Group A: 0.61 ± 0.19 Group B: 0.67 ± 0.07 Group C: 1.16 ± 0.54 Day 30: Group A: 0.66 ± 0.05 Group B: 1.10 ± 0.40 Group C: 1.24 ± 0.44 Day 60: Group A: 0.66 ± 0.06 Group B: 1.42 ± 0.41 Group C: 1.54 ± 0.41 Day 90: Group A: 0.66 ± 0.07 Group B: 1.15 ± 0.38 Group C: 1.63 ± 0.26 Statistical analysis revealed that there was a highly significant difference in Vitamin C levels in blood in Group C participants compared to Group A and Group B patients at all visits (*p* < 0.01)	The present study aimed at evaluating the antioxidant effect of oral administration of melatonin/ Vitamin E to chronic periodontitis patients who underwent scaling and root planing. Vitamin C was measured in the blood as it is a significant antioxidant with effects on the immune system that helps in preventing and resolution of periodontal disease. The results of elevated vitamin C in the blood of patients who underwent melatonin supplementation in contrast to Vitamin E and no supplementation reveals the biochemical effects of melatonin in the management of periodontal disease
8	Zare Javid A./2020/Iran	Randomized double-blinded placebo-controlled single-center trial with 2 groups The control group (22) Type 2 diabetes mellitus patients with severe symptoms of periodontal disease underwent non-surgical periodontal therapy (NSPT) including scaling and root planing with dental hygiene instructions followed by placebo tablet consumption for 8 weeks Intervention group (22) Type 2 diabetes mellitus patients with severe symptoms of periodontal disease underwent non-surgical periodontal therapy (NSPT) including scaling and root planing with dental hygiene instructions followed by melatonin tablet consumption for 8 weeks.	Melatonin 250 mg tablets were procured from Nature Made, USA, composed of 3 mg melatonin net, sodium starch glycolate, magnesium stearate Placebo 250 mg tablets were made in Ahvaz Jundishapur University containing cellulose, silicon dioxide, magnesium stearate, and starch with peppermint oil for flavor.	The Control group received NSPT followed by 2 placebo tablets once a day for 8 weeks to be consumed 1 h before bedtime. The intervention group received NSPT followed by 2 melatonin tablets once a day for 8 weeks to be consumed 1 h before bedtime.	Venous blood sample collected at baseline and 8 weeks post NSPT after 12 h overnight fasting. Sample centrifuged at 3000 g for 10 min at 4-degree centigrade and the serum was separated and stored at −70 degrees centigrade until further analysis.	IL 1 B, Malondialdehyde (MDA), Total antioxidant capacity (TAC), Superoxide dismutase (SOD), catalase (CAT), and glutathione peroxidase (GPx) were measured by spectrophotometric and ELISA methods in a reliable manner	Control group: IL-1B (pg/mL) Baseline: 2.47 ± 0.48 Post intervention: 2.33 ± 0.54 MDA (micromoles): Baseline: 17.49 ± 1.38 Post-intervention: 17.17 ± 1.39 TAC (millimoles): Baseline: 0.318 ± 0.06 Post intervention: 0.327 ± 0.08 SOD (units/mL): Baseline: 14.27 ± 2.52 Post-intervention: 14.49 ± 2.58 CAT (units/mL): Baseline: 23.14 ± 3.52 Post-intervention; 22.72 ± 5.58 GPx (units/mL): Baseline: 231.18 ± 67.28 Post-intervention; 233.18 ± 62.66 Intervention group: IL-1B (pg/mL) Baseline: 2.41 ± 0.55 Post intervention: 2.06 ± 0.48 MDA (micromoles): Baseline: 17.2 ± 1.82 Post-intervention: 16.13 ± 1.76 TAC (millimoles): Baseline: 0.289 ± 0.04 Post intervention: 0.313 ± 0.05 SOD (units/mL): Baseline: 13.91 ± 2.75 Post-intervention: 15.53 ± 4.37 CAT (units/mL): Baseline: 24.23 ± 4.54 Post-intervention; 27.47 ± 4.12 GPx (units/mL): Baseline: 243.04 ± 68.37 Post-intervention; 262.04 ± 62.45 Mean changes in the inflammatory and antioxidant markers post-intervention in both the groups: IL-1B: Control group: −0.14 ± 0.43 Intervention group: −0.34 ± 0.54 MDA: Control group: −0.31 ± 0.88 Intervention group: −1.07 ± 0.92 TAC: Control group: 0.009 ± 0.06 Intervention group: 0.02 ± 0.04 SOD: Control group: 0.21 ± 0.57 Intervention group: 1.61 ± 2.57 CAT: Control group: −0.41 ± 6.7 Intervention group: 3.23 ± 4.67 GPx: Control group: 2 ± 25.14 Intervention group: 19 ± 27.89 No significant difference in biochemical parameters observed between the 2 groups at baseline (*p* > 0.05) melatonin administration in the intervention group significantly reduced the levels of IL-1B and MDA at 8 weeks post-intervention compared to baseline (*p* < 0.05). This change was not observable in the control group. The mean levels of SOD, GPx, CAT, and TAC were elevated in the intervention group at 8 weeks post-baseline. These changes were significant compared to the control group (*p* < 0.05)	Increased oxidative stress and depleted antioxidants are a feature of periodontal disease and type 2 diabetes mellitus. Melatonin supplementation was found to significantly boost antioxidant levels and could mitigate the levels of inflammatory and oxidative stress markers The dose of 6mg in the present study for melatonin supplementation has a better effect on the biochemical markers than previous studies utilizing 2 mg, 3 mg, and 5 mg. The limitation of the study is the inadequate number of groups which should be planned in the future as follows. Group 1 (diabetes plus no periodontal treatment plus placebo), Group 2 (diabetes plus no periodontal treatment plus melatonin), Group 3 (diabetes plus NSPT plus placebo), Group 4 (diabetes plus NSPT plus melatonin)

## Data Availability

Not applicable.

## Supplementary Materials

The following are available online at https://www.mdpi.com/article/10.3390/ma14092417/s1, Table S1: Additional information on the included studies using topic melatonin formulation, Table S2: Additional information on the included studies using systemic melatonin formulation, Table S3: Summary of the RoBANS assessment of the included studies, Table S4: Summary of the RoB tool-based assessment of the included studies, Table S5. Summary of the SIGN 50 scorings of the included studies, Table S6. Summary of the GRADE scoring of the included studies.
