# Supplementary material for: Melatonin as a Topical/Systemic Formulation for the Management of Periodontitis: A Systematic Review
Source: Materials (Basel). 2021 May 6;14(9):2417. doi: 10.3390/ma14092417 (PMC8124881; doi:10.3390/ma14092417)
Supplement: Supplementary file 1 [file materials-14-02417-s001.zip › materials-1165162-supplementary.pdf]

materials

MDPI

Systematic Review

Melatonin as a Topical/Systemic Formulation for the Management of Periodontitis: A Systematic Review

Thodur Madapusi Balaji [1], Saranya Varadarajan [2], Raghunathan Jagannathan [3], Jaideep Mahendra [4], Hammam Ibrahim Fageeh [5], Hytham N. Fageeh [5], Shazia Mushtaq [6], Hosam Ali Baeshen [7], Shilpa Bhandi [8], Archana A. Gupta [9], A. Thirumal Raj [2], Rodolfo Reda [10], Shankaragouda Patil [11,*] and Luca Testarelli [10]

1 Department of Dentistry, Bharathiraja Hospital and Research Institute, Chennai 600017, India; tmbala81@gmail.com
2 Department of Oral Pathology and Microbiology, Sri Venkateswara Dental College and Hospital, Chennai 600130, India; vsaranya87@gmail.com (S.V.); thirumalraj666@gmail.com (A.T.R.)
3 Department of Periodontology, Tagore Dental College and Hospital, Chennai 600127, India; doctorraghunathan@gmail.com
4 Department of Periodontology, Meenakshi Ammal Dental College, Meenakshi Academy of Higher Education and Research, Chennai 600095, India; jaideep_m_23@yahoo.co.in
5 Department of Preventive Dental Science, College of Dentistry, Jazan University, Jazan 45412, Saudi Arabia; hafageeh@jazanu.edu.sa (H.I.F.); hfageeh@jazanu.edu.sa (H.N.F.)
6 Dental Health Department, College of Applied Medical Sciences, King Saud University, Riyadh 11362, Saudi Arabia; smushtaqdr@gmail.com
7 Department of Orthodontics, College of Dentistry, King Abdulaziz University, Jeddah 21589, Saudi Arabia; Habaeshen@kau.edu.sa
8 Department of Restorative Dental Sciences, Division of Operative Dentistry, College of Dentistry, Jazan University, Jazan 45412, Saudi Arabia; shilpa.bhandi@gmail.com
9 Department of Oral Pathology and Microbiology, Dr. D. Y. Patil Dental College and Hospital Dr. D. Y. Patil Vidyapeeth, Pune 411018, India; archanaanshumangupta@gmail.com
10 Department of Oral and Maxillofacial Sciences, Sapienza University of Rome, 00161 Rome, Italy; rodolforeda17@gmail.com (R.R.); luca.testarelli@uniroma1.it (L.T.)
11 Department of Maxillofacial Surgery and Diagnostic Sciences, Division of Oral Pathology, College of Dentistry, Jazan University, Jazan 45412, Saudi Arabia
* Correspondence: dr.ravipatil@gmail.com

Citation: Balaji, T.M.; Varadarajan, S.; Jagannathan, R.; Mahendra, J.; Fageeh, H.I.; Fageeh, H.N.; Mushtaq, S.; Baeshen, H.A.; Bhandi, S.; Gupta, A.A.; et al. Melatonin as a Topical/Systemic Formulation for the Management of Periodontitis: A Systematic Review. *Materials* **2021**, *14*, 2417. https://doi.org/10.3390/ma14092417

Academic Editors: Marco Annunziata, Carlo Bertoldi, Luigi Generali and Stefania Bergamini

Received: 15 March 2021
Accepted: 2 May 2021
Published: 6 May 2021

Publisher's Note: MDPI stays neutral with regard to jurisdictional claims in published maps and institutional affiliations.

Copyright: © 2021 by the authors. Licensee MDPI, Basel, Switzerland. This article is an open access article distributed under the terms and conditions of the Creative Commons Attribution (CC BY) license (http://creativecommons.org/licenses/by/4.0/).

Table S1. Additional information on the included studies using topic melatonin formulation.

S.No	First Author Name/Year of Publication/Country of Origin	Additional Information Extracted from the Included Studies
1	Antonio Cutando/2013/Spain	**Additional information**: healthy group (12 males, 18 females, age range:31–68 years, mean age: 47.0 ± 10.3 years), diabetic patients with periodontal disease (14 males, 16 females, age range:24–58 years, mean age: 43.1 ± 12.4 years). The healthy group had no diabetes mellitus and were periodontally healthy. The diabetic group had either type 1 or type 2 diabetes mellitus with advanced periodontitis. The diabetic group had 17 types of 1 diabetes mellitus and 13 types 2 diabetes mellitus patients. Diabetes was evaluated based on plasma fasting glucose values>126 mg/dL, casual blood glucose> 200 mg/dL and HbA1C values >6.5%. mean HbA1C values before the study was 8.43 ± 0.89. Periodontitis was evaluated by Florida probe periodograms in Community periodontal index-based teeth namely 17,16,11,26,27 and 46,31,36,37. All patients recruited had advanced periodontitis as per the above criteria. Exclusion criteria for the study were intake of bisphosphonates, melatonin altering drugs, oral contraceptives, antibiotic consumption and periodontal treatment for 6 months. Baseline findings: Group 1 (salivary readings of alkaline phosphatase(U/L): 7.34 ± 1.28, acid phosphatase (U/L): 20.55 ± 1.99, osteocalcin(ng/mL): 4.97 ± 1.35, osteopontin(microgram/mL): 2.44 ± 0.80), Group 2 (salivary readings of alkaline phosphatase(U/L): 40.51 ± 4.83, acid phosphatase (U/L): 83.08 ± 6.85, osteocalcin(ng/mL): 5.83 ± 1.41, osteopontin(microgram/mL): 12.49 ± 1.78). All parameters measured between group 1 and group 2 statistically significant ($p < 0.001$).
2	Antonio Cutando/2014/Spain	**Additional information**: healthy group (12 males, 18 females, age range:31–68 years, mean age: 47.0 ± 10.3 years), diabetic patients with periodontal disease (14 males, 16 females, age range:24–58 years, mean age: 43.1 ± 12.4 years). The healthy group had no diabetes mellitus and were periodontally healthy. The diabetic group had either type 1 or type 2 diabetes mellitus with advanced periodontitis. The diabetic group had 17 types of 1 diabetes mellitus and 13 types 2 diabetes mellitus patients. Method of diabetes assessment not mentioned. Periodontitis was evaluated by Florida probe periodograms and the gingival index was measured. All patients recruited had advanced periodontitis as per the above criteria. Exclusion criteria for the study were intake of bisphosphonates, melatonin altering drugs, oral contraceptives, antibiotic consumption, and periodontal treatment for 6 months. Baseline findings: Group 1 (salivary melatonin:4.5 ± 0.81pg/mL plasma melatonin:13.9 ± 3.87 pg/mL salivary RANKL:53.6 ± 42.94 pg/mL salivary OPG:20.3 ± 11.13 pg/mL), Group 2 (salivary melatonin:2.7 ± 0.81pg/mL plasma melatonin: 9.7 ± 3.27 pg/mL salivary RANKL:102.6 ± 66.67 pg/mL, salivary OPG:10.4 ± 7.61 pg/mL). All parameters measured between group 1 and group 2 statistically significant ($p < 0.001$)
3	Antonio Cutando/2015/Spain	**Additional information**: healthy group (12 males, 18 females, age range:31–68 years, mean age: 47.0 ± 10.3 years), diabetic patients with periodontal disease (14 males, 16 females, age range:24–58 years, mean age: 43.1 ± 12.4 years). The healthy group had no diabetes mellitus and were periodontally healthy. The diabetic group had either type 1 or type 2 diabetes mellitus with advanced periodontitis. The diabetic group had 17 types of 1 diabetes mellitus and 13 types 2 diabetes mellitus patients. Diabetes was evaluated based on plasma fasting glucose values>126 mg/dL, casual blood glucose> 200 mg/dL and HbA1C values >6.5%. mean HbA1C values before the study was 8.43 ± 0.89. Periodontitis was evaluated by Florida probe periodograms in Community periodontal index-based teeth namely 17,16,11,26,27 and 46,31,36,37. All patients recruited had advanced periodontitis as per the above criteria. Exclusion criteria for the study were intake of bisphosphonates, melatonin altering drugs, oral contraceptives, antibiotic consumption, and periodontal treatment for 6 months. Baseline findings: Group 1 salivary melatonin:4.5 ± 0.81pg/mL plasma melatonin:13.9 ± 3.87 pg/mL, TNF alpha: 0.82 ± 0.17 pg/mL, IL 6:0.38 ± 0.05 pg/mL, CRP: 0.21 ± 0.08 mg/L), Group 2(salivary melatonin: 2.7 ± 0.81 pg/mL plasma melatonin: 9.7 ± 3.27 pg/ mL, TNF alpha: 1.79 ± 0.19 pg/mL, IL 6: 0.57 ± 0.07 pg/mL, CRP: 0.39 ± 0.11mg/L). All parameters measured between group 1 and group 2 differed significantly ($p < 0.001$)
4	Javier Montero/2017/Spain	**Additional information:** Group 1(14 males, 16 females, age range:24–58 years, mean age: 43.1 ± 12.4 years, 17 type 1 Diabetes, 13, type 2 diabetes, mean HbA1C: 8.43 ± 0.89, baseline GCF IL 1 beta: 127.73 ± 99.50, IL 6: 0.57 ± 0.007, PGE2: 265.42 ± 101.6), Group 2 (13 males,17 females, age range: 29 to 59 years, mean age:45.46 ± 8.8 years, 12 type 1 diabetes, 18 type 2 diabetes, mean HbA1C: 7.7 ± 0.56, baseline GCF IL 1 beta: 122.47 ± 95.2, IL 6: 0.56 ± 0.007, PGE2: 263.45 ± 98.7), Group 3(12 males, 18 females, age range:31–68 years, mean age: 47.0 ± 10.3 years, baseline GCF IL 1 beta: 93.35 ± 59.26, IL 6: 0.38 ± 0.005, PGE2: 205.71 ± 118.09). Periodontitis was evaluated by Florida probe periodontograms in Community periodontal index-based teeth namely 17,16,11,26,27 and 46,31,36,37. Gingival index was measured additionally. Diabetes assessment and exclusion criteria not mentioned.

Table S2. Additional information on the included studies using systemic melatonin formulation.

S.No	First Author Name/Year of Publication/Country of Origin	Additional Information Extracted from the Included Studies
1	Marawar A.P/2014/India	Group A (80): periodontitis patients above the age of 18 years, gender and age details not provided, Group B(80): periodontitis patients above the age of 18, gender and age details not provided. Patients with cardiovascular disease, neurological disorder, metabolic and endocrine dysregulation, psychiatric, autoimmune, and liver dysfunction were excluded from the study. Gingival inflammation assessed by the gingival index (GI) in teeth numbers 16,21,24,36,41,44. Periodontal disease assessed by the Periodontal disease index (PDI) in indexed teeth namely, 16,21,24,36,41 and 44, and community periodontal index (CPI) were measured after dividing the mouth into sextants. Patients were randomized into 2 groups mentioned above. Details of randomization, method of patient allocation into the test or placebo groups, and details of blinding not mentioned. Clinical examination and indices mentioned measured at baseline, 30 days, 60 days, and 90 days
2.	Chitsazi M/2017/Iran	Additional information: A total of 60 subjects (29 females and 31 males) aged between 23–65 years (mean age 41 years) divided into 3 groups. All participants were systemically healthy. Inclusion criteria were the presence of moderate to severe chronic periodontitis with the presence of at least 3 pockets measuring 5–7 mm. Examination and diagnosis were performed by a single examiner who was blinded. Gingival index, Probing depth (PD) and Clinical attachment loss (CAL) assessed at baseline, 3 months, and 6 months following treatment. Exclusion criteria were prior use of non-steroidal anti-inflammatory drugs, antimicrobials up to 3 months before therapy, use of vitamin supplements and mouthwashes, smoking, recreational drug use, pregnancy, and lactation. Randomisation was done using randomization software.
3.	Hadi Bazyar/2019/Iran	A total of 96 types 2 diabetes mellitus patients were initially recruited into the study, after dental examination 46 patients were excluded and 50 patients were included after a thorough periodontal examination. The study was approved by the research ethics committee of the Ahvaz Jundishapur University (Ref. No.IR.AJUMS.REC.1395.685) and was registered in the Iranian Registry of Clinical Trials website (Ref no: IRCT2017011631993N1). Inclusion criteria were males and females, Body mass index of 18.5 to 30 kg/m2, confirmed diabetes mellitus type 2 with Fasting blood sugar > 126 mg/dL, HbA1C > 6.5% OR 2 h postprandial blood sugar > 200mg/dL. Periodontal disease was defined as mild and moderate periodontitis with probing depth > 4mm and clinical attachment loss between 1–4mm measured with a UNC 15 periodontal probe at 6 sites per tooth in addition to the presence of bleeding on probing. Exclusion criteria were kidney failure, pregnancy, breastfeeding, thyroid disease, traveling history of 2 weeks and above, smoking, intake of immunosuppressive medication, insulin, antibiotics, presence of severe periodontitis, intake of vitamins, antioxidants, and anti-inflammatory agents. The 50 chosen patients were allocated into the control or intervention group by a randomly permuted block procedure. In this method, 2 separate codes A and B were used to generate six groups with block design AABB, BBAA, ABAB, BABA, ABBA, BABA. The coding plan was done by a person unconnected to the study. All the patients included in the trial underwent anthropometric measurements such as height, weight, BMI, waist circumference, waist to hip ratio by a professional nutritionist, and a 3-day 24-h dietary recall and assessment by nutritionist 4 software. The subjects were advised to maintain their current diet plan through the study. The physical activity of the recruited patients was evaluated by a questionnaire consisting of 3 parts including heavy activity with a coefficient of 4and walking with a coefficient of 3.3. Light activity ranged from 0 to 600 min, moderate activity ranged from 600 to 3000 min, and heavy activity over 3000 min. A continuous minimum of 10 min of activity performance was considered essential to score the patients. Based on the above criteria, the control group had the following variables and parameters (Mean age: 51.45 ± 5.03 years, 16 females/6 males, mean height: 162.95 ± 9.27 cms, mean waist circumference: 102.04 ± 8.69cms, mean hip circumference: 107.18 ± 8.08 cms, mean waist-hip ratio: 0.95 ± 0.08, mean physical activity: 320.86 ± 170.58 min), The intervention group had the following variables and parameters (mean age: 53.72 ± 6.68 years, 14 females/8 males, mean height: 164.4 ± 6.7 cms, mean waist circumference: 101.22 ± 9.99 cms, mean hip circumference: 106.59 ± 9.7 cms, mean waist-hip ratio: 0.95 ± 0.05, mean physical activity: 293.31 ± 172.15 min). The variables and parameters measured were not statistically significant between the 2 groups ($p > 0.05$). Nutritionist software measured values were generated for energy, carbohydrate, protein, fat, cholesterol, saturated fat, Vitamin A, Beta carotene, Selenium, Vitamin C, alpha-tocopherol, Vitamin E intake per day for the 2 groups. These values were not significantly different in both the groups at baseline and 8 weeks post-intervention (p>0.05). Both patients and researchers were blinded in this study. Out of 50 patients recruited, 3 patients in the test group and 3 patients in the placebo group discontinued the study after baseline treatment and did not do the follow-up visits
4.	Hesham-El-Sharkawy/2019/Egypt	Initially 182 patients were examined for eligibility for the study. Out of them, 102 individuals were excluded as 75 subjects did not meet the inclusion criteria and 27 subjects refused to participate. Hence after the final exclusion, A total of 80 patients diagnosed with generalized chronic periodontitis and primary insomnia were recruited for the study initially after obtaining informed consent. They were included either in the melatonin or placebo group with a 1:1 allocation ratio. 2 participants from the melatonin group and 4 participants from the placebo group were lost during the follow-up phase. Hence a total of only 74 patients participated in the

		randomized controlled clinical trial. The randomized control trial was conducted following the Helsinki declaration in 2013 and approved by the institutional review board of Mansoura University, Egypt (18020118). The CONSORT guidelines were followed in this trial after official registration with ClinicalTrials. Under the name DENT-2017 with identification number NCT03368430. Patient recruitment was done between June 2016 and July 2017. A questionnaire was prepared in Arabic language and was used for patient recruitment. The Athens-Insomnia score (AIS) was used to diagnose sleep disorder and patients with AIS score>6 were recruited into the study. Also, the selected patients were chosen in a way that in addition to insomnia they had a minimum of 20 teeth and were diagnosed to have moderate to severe chronic periodontitis ie, radiographic evidence of bone loss, and presence of pocket depth >5mm and at least 3 sites in each quadrant with loss of attachment of >4mm. exclusion criteria were diabetes mellitus, smokers, patients with night shifts, patients with cancer, autoimmune diseases, osteoporosis, patients using antibiotics and anti-inflammatory drugs for the past 3 months, and history of periodontal therapy within 1 year were excluded. The randomization of the patients into the melatonin/placebo group was accomplished by computer-generated randomization and melatonin/placebo was given in sealed coded bottles which were not known to the patients or operators. No adverse reactions were observed during the trial. headache, dizziness, nausea, constipation, and abdominal cramp was recorded in 0 to 2 cases in the melatonin group. Patients In the melatonin and placebo group showed drug compliance of 90.4% and 87.5% respectively. Demographic data of the Group 1(mean age: 45.6 ± 7.1, gender:21M/17F, AIS score: 8.4 ± 1.1, Teeth22.1 ± 2.4:, Plaque index(PI): 2.3 ± 0.5, Gingival index(GI): 2.1 ± 0.6, Bleeding on probing: 63 ± 21, Probing depth(PD): 4.3 ± 0.8, Clinical attachment level(CAL): 4.8 ± 0.9), Group 2(mean age: 46.7 ± 8.3, gender:20M/16F, AIS score: 8.7 ± 1.2, Teeth: 23 ± 2.2, Plaque index(PI): 2. 4 ± 0.7, Gingival index(GI): 2.2 ± 0.4, Bleeding on probing: 59 ± 19, Probing depth(PD): 4.4 ± 0.7, Clinical attachment level(CAL): 4.7 ± 1.0). the clinical parameters were not significantly different between the groups (p>0.05)
**5.**	Marawar A.P/2019/India	A total of 160 patients with chronic periodontitis were recruited for the study. The study was approved by the institutional ethical committee of the Pravara Institute of Medical sciences. The total period of study was 1 year. Patients with chronic periodontitis were recruited into the study after obtaining informed consent. No details of diagnostic criteria to ascertain chronic periodontitis have been mentioned. The details of probing depth and clinical attachment levels and other periodontal parameters are not mentioned. The exclusion criteria for the study were postoperative patients, patients on night duty jobs, drivers, and heavy machine technicians, pregnant women, lactating mothers, patients with significant systemic diseases on medication for the same. No method of randomization or grouping of patients has been described. Group A comprised of 71.25% males and 28.75% females and group B comprised of 56.25% males and 43.75% females. Numerical values are not provided for the age of participants. Only bar diagram
**6.**	Manuel Tinto/2020/Italy	The study followed CONSORT 2010 guidelines and was conducted at the Periodontal unit of Santa Apollonia Dental Center. The study was approved by the local ethics committee (ASST Monza e della Brianza, Monza, Italy). All patients received written informed consent. This study was conducted as a monocentric, randomized, placebo-controlled, triple-blind clinical trial. The medication was delivered in anonymous blister packs to the blinded clinical investigator and blinded patient by the pharmacy. The study was organized into 4 different phases 1. Enrolment 2. Treatment 3. 6 months follow up evaluation 4. Data elaboration and analysis. The enrollment phase included screening, evaluation of patient's eligibility, medical history, full mouth scoring of probing depth (PD), full mouth bleeding scores (FMBS%), full mouth plaque scores (FMPS%) at 6 sites per tooth. The inclusion criteria for the study were healthy adult patients between 30 and 70 years, with untreated severe stage 3 (interdental clinical attachment loss >5mm, less than 4 teeth lost, maximum PD > 6mm), according to the definition of World Workshop of periodontics, 2017. Exclusion criteria were smoking > 20 cigarettes per day, uncontrolled diabetes, immunosuppression, current therapy with antiresorptive drugs, pregnancy, breastfeeding, need for antibiotic therapy, and therapy with mood modulators or sedatives. Complete periodontal charting was performed at baseline and 6 months. PD change was considered the primary outcome with 2 subgroups one having probing depth of 4–5mm and the other having probing depth>6mm. Secondary outcomes were mean PD, FMPS%, FMBS% changes. Concerning blinding participants and operators were blinded from beginning to the end of the experiment. As earlier described 20 patients afflicted by stage 3 periodontitis were included in the study. 12 males and 8 females formed the study population with a mean age of 45.6 years. No drop out was observed in the study. Melatonin was well tolerated by all the participants. While 10 % of the patients had a headache, 20% had sleepiness but the adverse effects did not affect their routine.
**7.**	Marwar A.P/2020/India	The present study was performed on a total of 240 patients. It was approved by the institutional ethical committee of Pravara Institute of Medical Sciences, Loni, Ahmednagar, Maharashtra, India. The study was carried out between January to December 2008. Patients of chronic periodontitis aged between 18–65 years were recruited for the study. No details about pocket depth, attachment level, and other periodontal parameters are mentioned. Exclusion criteria were postoperative patients, patients having night duties, drivers, heavy machine workers, pregnant women, lactating mothers, patients on drugs, and with clinically evident systemic disease. Group A consisted of 71.25% males and 28.75% females. Group B consisted of 58.75% males and 41.25% females. Group C consisted of 56.25% males and

		43.75% females. Numerical values are not provided for the age of participants. Only bar diagram depicted which does not provide actual values.
**8.**	Zare Javid A./2020/Iran	A total of 96 types 2 diabetes mellitus patients were initially recruited into the study, after dental examination 46 patients were excluded and 50 patients were included after the thorough periodontal examination. The study was approved by the research ethics committee of the Ahvaz Jundishapur University (Ref. No.IR.AJUMS.REC.1396.157) and was registered in the Iranian Registry of Clinical Trials website (Ref no: IRCT2017030831993N4). Inclusion criteria were males and females, Body mass index of 18.5 to 30 kg/m2, confirmed diabetes mellitus type 2 with Fasting blood sugar>126 mg/dL, HbA1C > 6.5 %- OR 2-h postprandial blood sugar>200mg/dL. Periodontal disease was defined as mild and moderate periodontitis with probing depth>4mm and clinical attachment loss between 1-4mm. measured with a UNC 15 periodontal probe at 6 sites per tooth in addition to the presence of bleeding on probing. Exclusion criteria were kidney failure, pregnancy, breastfeeding, thyroid disease, traveling history of 2 weeks and above, smoking, intake of immunosuppressive medication, insulin, antibiotics, presence of severe periodontitis, intake of vitamins, antioxidants, and anti-inflammatory agents. The 50 chosen patients were allocated into control or intervention by a random block permutation procedure, block analysis combined analysis. All the patients included in the trial underwent anthropometric measurements such as height, weight, BMI, waist circumference, waist to hip ratio by a professional nutritionist, and a 3-day 24-h dietary recall and assessment by nutritionist 4 software. The subjects were advised to maintain their current diet plan throughout the study. The physical activity of the recruited patients was evaluated by a questionnaire consisting of 3 parts including heavy activity with a coefficient of 4and walking with a coefficient of 3.3. Light activity ranged from 0 to 600 min, moderate activity ranged from 600 to 3000 min, and heavy activity over 3000 min. A continuous minimum of 10 min of activity performance was considered essential to score the patients. Based on the above criteria, the control group had the following variables and parameters (Mean age: 51.45 ± 5.03 years, 16 females/6 males, mean height: 162.95 ± 9.27 cms, mean waist circumference: 102.04±8.69cms, mean hip circumference: 107.18 ± 8.08 cms, mean waist-hip ratio: 0.95±0.08, mean physical activity: 320.86±170.58 min), The intervention group had the following variables and parameters (mean age: 53.72±6.68 years, 14 females/8 males, mean height: 164.4 ± 6.7 cms, mean waist circumference: 101.22 ± 9.99 cms, mean hip circumference: 106.59 ± 9.7 cms, mean waist-hip ratio: 0.95 ± 0 .05, mean physical activity: 293.31 ± 172.15 min). The variables and parameters measured were not statistically significant between the 2 groups(p>0.05). Nutritionist software measured values were generated for energy, carbohydrate, protein, fat, cholesterol, saturated fat, Vitamin A, Beta carotene, Selenium, Vitamin C, alpha-tocopherol, Vitamin E intake per day for the 2 groups. These values were not significantly different in both the groups at baseline and 8 weeks post-intervention (p>0.05). Only 44 subjects, 22 in each group finally completed the study.

Table S3. Summary of the RoBANS assessment of the included studies.

S.No.	First Author Name/Year of Publication/Country of Origin	Selection of Participants	Confounding Variables	Measurement of Intervention (Exposure)	Blinding for Outcome Assessment	Incomplete Outcome Data	Selective Outcome Reporting
1.	Antonio Cutando/2013/Spain	Yes/Low (Age and gender distribution mentioned and taken into account)	Yes/High (diabetes mellitus is a confounder for melatonin levels and periodontal disease)	Yes/Unclear (details not provided for the placebo group)	Yes/High (no blinding was done)	Unclear (results of the study not reported for the placebo group, attrition of participants not mentioned)	Yes/High (improvement with application reported only in the test group, no reporting for the placebo group)
2.	Antonio Cutando/2014/Spain	Yes/Low (Age and gender distribution mentioned and taken into account)	Yes/High (diabetes mellitus is a confounder for melatonin levels and periodontal disease)	Yes/Unclear (details not provided for the placebo group)	Yes/High (no blinding was done)	Yes/Unclear (results of the study not reported for the placebo group, attrition of participants not mentioned)	Yes/High (improvement with application reported only in the test group, no reporting for the placebo group)
3.	Antonio Cutando/2015/Spain	Yes/Low (Age and gender distribution mentioned and taken into account)	Yes/High (diabetes mellitus is a confounder for melatonin levels and periodontal disease)	Yes/Unclear (details not provided for the placebo group)	Yes/High (no blinding was done)	Yes/Unclear (results of the study not reported for the placebo group, attrition of participants not mentioned)	Yes/High (improvement with application reported only in the test group, no reporting for the placebo group)
4.	Javier Montero/2017/Spain	Yes/Low (Age and gender distribution mentioned and taken into account)	Yes/High (diabetes mellitus is a confounder for melatonin levels and periodontal disease)	Yes/Unclear (details not provided for the systemically healthy placebo group)	Yes/High (no blinding was done)	Yes/Unclear (results of the study not reported for the systemically healthy placebo group, attrition of participants not mentioned)	Yes/High (improvement with application reported the only test versus placebo group concerning diabetic patients with periodontitis, no reporting for the systemically healthy placebo group)
5.	Marawar A.P/2019/India	Yes/High (selection details not provided adequately concerning periodontal disease diagnosis and other periodontal parameters	Yes/High (no mention of mean age of the groups as numerical values done as age is a confounder for melatonin levels and periodontal disease)	Yes/Low (exposure data provided for both groups)	Yes/High (no blinding was done)	Yes/Low (outcome measures reported satisfactorily)	Yes/High (changes in periodontal parameters with/without melatonin not taken into account or mentioned)
6.	Marwar A.P/2020/India	Yes/High (selection details not provided adequately concerning periodontal disease diagnosis and other periodontal parameters	Yes/High (no mention of mean age of the groups as numerical values done as age is a confounder for melatonin levels and periodontal disease)	Yes/Low (exposure data provided for all the 3 groups)	Yes/High (no blinding was done)	Yes/Low (outcome measures reported satisfactorily)	Yes/High (changes in periodontal parameters with/without melatonin not taken into account or mentioned)

Table S4. Summary of the RoB tool-based assessment of the included studies.

S.No.	First Author Name/Year of Publication/Country of Origin	Random Sequence Generation	Allocation Concealment	Blinding of Participants & Personnel	Blinding of /Outcome Assessment	Incomplete Outcome Data Addressed	Free of Selective Reporting	Free from Other Bias
1.	Marawar A.P/2014/India	Yes/Unclear (not reported)	Yes/Unclear (not reported)	Yes/Unclear (Not reported)	Yes/Unclear (Not reported)	Yes/Unclear (not mentioned)	Yes/Low (all periodontal indices measured have been reported at baseline and post-intervention	Yes/High (age and gender distribution not mentioned which could cause selection bias)
2.	Chitsazi M/2017/Iran	Yes/Low (randomization was done using commercially available software)	Yes/High (no placebo used despite mentioning as a randomized controlled clinical trial, no allocation concealment mentioned)	Yes/High (only one examiner who diagnosed the patients at baseline was masked and blinded)	Yes/High (examiner who assessed the outcome was not blinded)	Yes/Low (all data relevant to participants fully presented, no incomplete data)	Yes /Low (all periodontal indices and parameters measured have been reported at baseline and post-intervention.)	Yes/High (selection bias concerning the diagnosis of periodontal disease)
3.	Hadi Bazyar/2019/Iran	Yes/Low (randomization was done using a random block permutation procedure	Yes/Low (test and placebo tablets were matched and patients were unaware of allocation and blinded)	Yes/Low (double-blinded study with patients and researchers blinded)	Yes/Low (double-blinded study with patients and researchers blinded)	Yes/Low (Incomplete data addressed in terms of patient attrition)	Yes/Low (all periodontal indices, parameters, and markers measured have been reported at baseline and post-intervention.)	Yes/High (Diabetes mellitus is a potential confounder for melatonin levels and periodontal disease burden. No control groups have been implemented)
4.	Hesham-El-Sharkawy/2019/Egypt	Yes/Low (Randomisation was done using software)	Yes/Low (test and placebo tablets were sealed and coded and patients were unaware of allocation and blinded)	Yes/Low (double-blinded study with patients and researchers blinded)	Yes/Low (double-blinded study with patients and researchers blinded)	Yes/Low (Incomplete data addressed in terms of patient attrition)	Yes/Low (all periodontal indices, parameters, and markers measured have been reported at baseline and post-intervention.)	Yes/High (the use of chlorhexidine mouthwash following scaling and root planing could be a potential confounder damping the effects of melatonin)
5.	Manuel Tinto/2020/Italy	Yes/Low (Randomisation was done in the study)	Yes/Low (test and placebo tablets were packed in anonymous bister packs and given to the patient's post-randomization)	Yes/Low (double-blinded study with patients and researchers blinded)	Yes/Low (double-blinded study with patients and researchers blinded)	Yes/Low (lack of dropouts mentioned in the study)	Yes/High (only probing depth changes mentioned, data about full mouth plaque and bleeding scores not presented)	Yes/Low (no other bias identified)
6.	Zare Javid A./2020/Iran	Yes/Low (randomization was done using a random block permutation procedure	Yes/low (test and placebo tablets were matched and patients were unaware of allocation and blinded)	Yes/low (double-blinded study with patients and researchers blinded)	Yes/low (double-blinded study with patients and researchers blinded	Yes/ low (Incomplete data addressed in terms of patient attrition)	Yes /low (all periodontal indices, parameters, and markers measured have been reported at baseline and post-intervention.)	Yes/high (Diabetes mellitus is a potential confounder for melatonin levels and periodontal disease burden. No control groups have been implemented)

Table S5. Summary of the SIGN 50 scorings of the included studies.

S.No	First Author Name/Year of Publication/Country of Origin	Clear Focus Question	Randomization Performed Adequately	Allocation Concealment	Blinding of Patients and Personnel	Homogeneity of Cases and Controls	The Difference between the Groups is Only the Treatment in Concern	Outcome Measures Standardized	Dropouts Assessed if Present	Intention Treat Analysis (All Subjects Analyzed in the Group Where They Belong To)	The Similarity of Data if the Study is Multicentric	Score
1.	Marawar A.P/2014/India	Yes	no	no	no	no	no	yes	yes	yes	Not applicable	-
2.	Chitsazi M/2017/Iran	yes	yes	no	no	no	no	yes	yes	yes	Not applicable	-
3.	Hadi Bazyar/2019/Iran	yes	yes	yes	yes	no	no	yes	yes	yes	Not applicable	+
4.	Hesham-El-Sharkawy/2019/Egypt	yes	yes	yes	yes	no	no	yes	yes	yes	Not applicable	+
5.	Manuel Tinto/2020/Italy	yes	yes	yes	yes	no	no	yes	yes	yes	Not applicable	+
6.	Zare Javid A./2020/Iran	yes	yes	yes	yes	no	no	yes	yes	yes	Not applicable	+

Notes: ++ indicates all the criteria were well addressed; + indicates if 1–3 criteria were poorly addressed; - indicates if >3 criteria of the study were poorly addressed.

Table S6. Summary of the GRADE scoring of the included studies.

S.No	First Author Name/Year of Publication/Country of Origin	Confidence in the Estimate of Effect GRADE 1	The Magnitude of the Estimate of Effect GRADE 2	Safety GRADE 3	Strength of the Recommendation GRADE 4
1.	Antonio Cutando/2013/Spain	C	ND	+1	Weak, in favor
2.	Antonio Cutando/2014/Spain	C	ND	+1	Weak, in favor
3.	Marawar A.P/2014/India	C	ND	+1	Weak, in favor
4.	Antonio Cutando/2015/Spain	C	ND	+1	Weak, in favor
5.	Javier Montero/2017/Spain	C	ND	+1	Weak, in favor
6.	Chitsazi M/2017/Iran	C	ND	+1	Weak, in favor
7.	Hadi Bazyar/2019/Iran	B	ND	+1	Weak, in favor
8.	Hesham-El-Sharkawy/2019/Egypt	B	ND	+1	Weak, in favor
9.	Marawar A.P/2019/India	C	ND	+1	Weak, in favor
10.	Manuel Tinto/2020/Italy	B	ND	+1	Weak, in favor
11.	Marwar A.P/2020/India	C	ND	+1	Weak, in favor
12.	Zare Javid A./2020/Iran	B	ND	+1	Weak, in favor

Note: GRADE 1: A indicates further research is very unlikely to change confidence in the estimate of effect; B indicates further research is likely to have an important impact on confidence in the estimate of effect and may change the estimate; C indicates further research is very likely to have an important impact on confidence in the estimate of effect and is likely to change the estimate; D indicates any estimate of effect is very uncertain.

GRADE 2: Report effect size were categorized as none (<0.2), small (0.2–0.5), moderate (0.5–0.8), large (>0.8) or not described (ND).

GRADE 3: +2 indicates safe with infrequent adverse events and interactions; +1 indicates relatively safe but with frequent but no serious adverse events and interactions; 0 indicates safety not well understood or conflicting; −1 indicates has safety concerns that include infrequent but serious adverse events and/or interactions; −2 indicates serious safety concerns that include frequent and serious adverse events and/or interactions.

Recommendation: Based on GRADE 1, 2, and 3, the recommendation was made as follows:

A strong recommendation in favor of or against (very certain that benefits do, or do not, outweigh risks and burdens); no recommendation; weak recommendation in favor of or against (benefits and risks and burdens are finely balanced, or appreciable uncertainty exists about the magnitude of benefits and risks).
